# Mapping global eco-environment vulnerability due to human and nature disturbances

**DOI:** 10.1016/j.mex.2019.03.023

**Published:** 2019-04-11

**Authors:** Kim-Anh Nguyen, Yuei-An Liou

**Affiliations:** a Center for Space and Remote Sensing Research, National Central University, 300, Jhongda Rd., Jhongli District, Taoyuan City 32001, Taiwan, ROC; b Taiwan Group on Earth Observations, Hsinchu, Taiwan, ROC; c Institute of Geography, Vietnam Academy of Science and Technology, 18 Hoang Quoc Viet Rd., Cau Giay, Hanoi, Viet Nam

**Keywords:** GIS framework for mapping eco-environmental vulnerability, Anthropogenic stress, Natural variation, Assessment framework, Environmental sustainability

## Abstract

Identifying vulnerable levels of eco-environment over a global scale is critical for environmental management and ecological conservation. We present the method to optimize the use of freely assessable datasets to derive 16 factors for a proposed assessment framework (Nguyen and Liou, 2019; Liou et al., 2017; Nguyen et al., 2016) [[1], [2], [3]]. Results show that the datasets are suitable for evaluating global eco-environmental vulnerability (GEV). PM_2.5_ that is a hazardous substance in environment and an anthropogenic disturbance associated with nature and human-made influence is selected to validate the GEV map. The GEV map well correlates with PM_2.5_ distribution patterns with correlation coefficient of approximately 0.82. All datasets and mapping procedures are processed in ArcGIS 10.3/QGIS 2.16.3 software. Advantages of our method include three aspects:

• The analysis procedure is simple but powerful, while dealing with various complex environmental issues.
• The framework is flexible to adjust influential indicators subject to the conditions of concerned regions and purposes of decision makers.
• The framework can be easily applied for different concerned regions over various scales.

Our findings include GEV mapping and eco-protection zoning that provide key hotspots of eco-environmental vulnerability levels over a global scale for the decision makers and people to take further actions to lessen disturbances and achieve environmental sustainability.

## Graphical abstract

**Figure:**
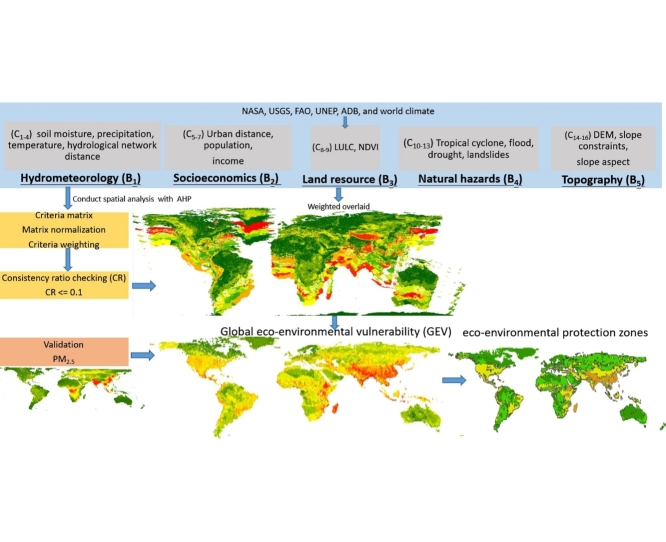

**Specifications Table**

**Table:** 

Subject Area:	• Earth and Planetary Sciences • Environmental Science
More specific subject area:	Describe narrower subject area Environment is disturbed by anthropogenic stress and natural variation
Method name:	GIS framework for mapping eco-environmental vulnerability
Name and reference of original method:	If applicable, include full bibliographic details of the main reference(s) describing the original method from which the new method was derived. The current method [1] can be considered as an extended work of our previous research outcomes [2,3] where we performed eco-environmental vulnerability assessment (EVA) at a provincial scale of Vietnam. The innovation in our current research [1] is to provide the first global-scale map of eco-environmental vulnerability. The six global environmental vulnerability (GEV) categories and their map of global “hotspots” are novel and will be proven highly useful to researchers and decision makers around the world working on the issues of sustainability, conservation, development, and vulnerability. Subsequently, three eco-environmental zones are introduced with functions and advices for activities and planning for the regions of concern. Considering both natural and manmade disturbances, GEV analysis can be of significant value in (i) Enriching the guidance of global and regional planning and construction, and protection of the ecological environment; (ii) Harmonizing information from the reports that employ different approaches or definitions; (iii) Providing a feasible framework template of EVA, which benefits environmental education; and (iv) Conveying information to the public for enhancing the role of communities in solving environmental problems. [1] Nguyen, K.A, Y.-A. Liou*, 2019. Global mapping of eco-environmental vulnerability from human and nature disturbances. Science of the Total Environment, 664 (2019) 995–1004. doi.org/10.1016/j.scitotenv.2019.01.407 [2] Y.A. Liou, K.A Nguyen*, and M.H. Li., 2017. Assessing spatiotemporal eco-environmental vulnerability by Landsat data, Ecological Indicators. Volume 80, September 2017, Pages 52–65. DOI.org/10.1016/j.ecolind.2017.04.055. [3] Nguyen, K.A, Y.A. Liou*, M.H. Li, and T.A. Tran., 2016. Zoning eco-environmental vulnerability for environmental management and protection. Ecological Indicators, Vol 69, Pages 100–117. DOI: 10.1016/j.ecolind.2016.03.026
Resource availability:	If applicable, include links to resources necessary to reproduce the method (e.g. data, software, hardware, reagent)

## Materials

Materials used in the study including (1) soil moisture, (2) precipitation, (3) temperature, (4) hydrological network, (5) population, (6) income, (7) land use/land cover, (8) Normalized Difference Vegetation Index (NDVI), (9) natural hazards (tropical cyclones, landslides, flood, drought), (10) Digital Elevation Model (DEM), and (11) PM_2.5_ for validation. All details of these materials are described in Table 1 and data preparation step 1. Software packages used include ArcGIS and QGIS.

**Table 1:** Indicators used to evaluate GEV including their sources, data description, and preparation, and a brief explanation of their roles.

Major disturbance determinants	Indicators	Role in environment profile	Sources
Hydrometeorology (B_1_)	Soil moisture (C_1_)	Soil moisture is vitally important in controlling the exchange of water and heat energy between land surface and atmosphere through evapotranspiration and as a key variable to define flood control, soil erosion, and slope failure.	Moran et al., (2007) [4]
	Precipitation (C_2_)	Precipitation is important for soil and plant growth and useful for determination of weather patterns regarding to early warning of drought and flood.	Ficka and Hijmans (2017) [5]
	Temperature (C_3_)	Average global air temperature is useful to classify weather patterns in combination with precipitation and soil moisture.	Ficka and Hijmans (2017) [5]
	Distance from hydrological network (C_4_)	Availability of surface water is important for environment especially in urban cities for cooling heat island effect.	http://wp.geog.mcgill.ca/hydrolab/ [6]
Socioeconomics (B_2_)	Population (C_5_)	Population plays an important role in eco-environmental vulnerability assessment since it contributes to determine human pressure on eco-environment. In general, more people and higher population density likely cause heavier pressure on environment resulting in higher vulnerability.	https://data.worldbank.org [7]
	Income (C_6_)	This indicator shows average income of each country from high to low income (highly-developed countries to developing countries). In general, in the developing countries, the eco-environment is likely to be disturbed more than developed countries since they are on the fast growing processes of urbanization and industrialization. Income also reflects the education level as well as public awareness of eco-environmental protection.	https://data.worldbank.org [7]
	Distance from urbanized areas (C_7_)	This indicator determines the influence from the urban by spatial distance. Exposure from urban affected the eco-environment by the stress from the city like pollution from vehicles and air-condition, and trash from households, and wastewater. It is likely that the farther from the urban the better the eco-environment.	http://preview.grid.unep.ch [8]
Land resource (B_3_)	Land use/land cover (LULC) (C_8_)	LULC is an important determinant of eco-environmental vulnerability due to its contribution to and general influence on environmental quality. The areas without or with less vegetation cover are more vulnerable than the dense vegetation areas. Impervious surface materials conserve more heat during the day and release it more slowly at night than natural materials like soil or vegetation.	https://reverb.echo.nasa.gov [9] https://landcover.usgs.gov/glc/ [10]
	Normalized Difference Vegetation Index (NDVI) (C_9_)	NDVI is a crucial indicator to measure the greenness of vegetation and vegetation plays an important role in maintaining good eco-environment. Regions that are less or without vegetation may cope with higher vulnerability.	https://reverb.echo.nasa.gov [9]
Natural hazards (B_4_)	Drought (C_10_)	These indicators determine the areas constantly affected by natural hazards resulting in environmental decline.	Global Risk Data Platform http://preview.grid.unep.ch [8]
	Tropical cyclones (C_11_)		
	Landslides (C_12_)		
	Flood (C_13_)		
Topography (B5)	DEM (C_14_)	DEM plays an important role in defining topographic condition, determining the features of land surface such as incoming solar radiation, tree types, and potential exposure to hazards like landslide, and drought.	http://glcf.umd.edu/data/srtm/ [11]
	Slope constraint (C_15_)	Slope constraint is a factor influencing land-use decision and the item “Land utilization possibilities”. The influence of terrain on erosion is great important. Steeper slopes are also associated with shallower soils in general and with a higher risk for soil degradation and landslides [[13], [14], [15]].	http://www.fao.org/geonetwork [12]
	Slope aspect (C_16_)	Slope aspect and topographic position contribute to define annual mean temperature, potential energy incoming and evapotranspiration. Resulting in vegetation structure, ground moisture, snow retention, plant communities and surface temperature are all characteristics influenced by aspect [16].	SRTM DEM http://glcf.umd.edu/data/srtm/ [12]
Anthropogenic stress and natural influence	PM_2.5_	An independent variable PM_2.5_ that can be considered as an anthropogenic disturbance associated with nature and human-made influence is chosen to validate the GEV map	http://sedac.ciesin.columbia.edu [17]

## Method details

We propose a framework for evaluation of eco-environmental vulnerability at global scale by using GIS techniques and freely accessible datasets. However, our method and framework can be applied not only to any environmental issues due to any specific natural stresses such as forest fire, typhoon, flooding, drought or anthropogenic disturbances such as pollution, industrialization, urbanization, but also to any region of concern. There are many influential factors that can affect eco-environment. Choosing the right set of indicator and mapping procedure are crucial for achieving meaningful and reliable quantitative results.

In this study we extend our previous works conducted over a regional scale at Thua Thien-Hue Province of Vietnam [2,3] by upscaling the assessment framework. The upscaling was performed by considering influential factors retrieved from global datasets across five domains (natural hazards, society-economics, hydrometeorology, and topography and land resource) that aim to visualize the nature and human disturbances on eco-environmental vulnerability. Nguyen et al. (2016) proposed an assessment framework to evaluate the eco-environmental vulnerability in association with 16 variables with six of them constructed from Landsat 8 satellite image products. 16 variables were taken into account and organized into four domains: (1) hydro-meteorology, (2) social-economics, (3) land resource, and (4) topography. This framework is relied on remote sensing data, digital maps and in situ measurements with aid of AHP and GIS. In this framework, the weights are stressed on society-economics and topography (0.329) followed by hydrometeorology (0.2) and land resource (0.142). Details of weights and applied AHP process were given in Table 1 and appendix of Nguyen et al. (2016). From a long-term point of view to monitor spatiotemporal eco-environment, it often becomes a barrier by using in situ measurements because of their limited spatiotemporal resolution and insufficient historical data. Thus, Liou et al. (2017) revised the assessment framework that mainly relied on variables, which can be retrieved from time-series Landsat satellite images. This improved framework is more suitable for long-term eco-environmental monitoring and resolved the difficulties in obtaining long-term in situ measurement. In addition, the framework proposed by Liou et al. (2017) allowed to capture both natural and manmade attributes to some extent given place and time, such as the impacts of land use, land cover change and urbanization on eco-environment. In these works of regional scale, all variables/factors were converted into a grid format with resolution of 100 m × 100 m and set up in the coordinate of WGS 84, zone 48 north in the ArcGIS version 10.2.

Globally speaking, it is almost infeasible to process all the data and variables at uniformed 100 m of resolution when, in particular, input variables cannot be derived from one kind of dataset or single satellite image as Liou et al. (2017) did. Thus, for a study over a globe scale, we further improved the assessment framework. Totally, 16 indicators were derived from ten global datasets (Table 1) and one global dataset of PM_2.5_ was used for validation. In the global improved framework, we considered one more domain, i.e., natural hazards. In this domain, we took into consideration of four kinds of dominated hazards, including flood, drought, landslide and cyclones. The influence of the four kinds hazards were considered equally with weight of 0.25. Thus, the global weight was revised in comparison with the previous framework with criteria that can capture natural and human disturbance, as presented in Table 3 with summary given in the diagram in Fig. 2. All indicators are computed or converted and resampled to be of same GEOtiff format and resolution of a pixel size 0.0833 × 0.0833°. The size of the globe for this resolution contains approximately 4320 columns and 1673 rows. All GEOtiff raster files were projected to spatial reference of WGS-84. Processing steps were mainly performed in ArcGIS 10.3 environment.

**Fig. 1:**
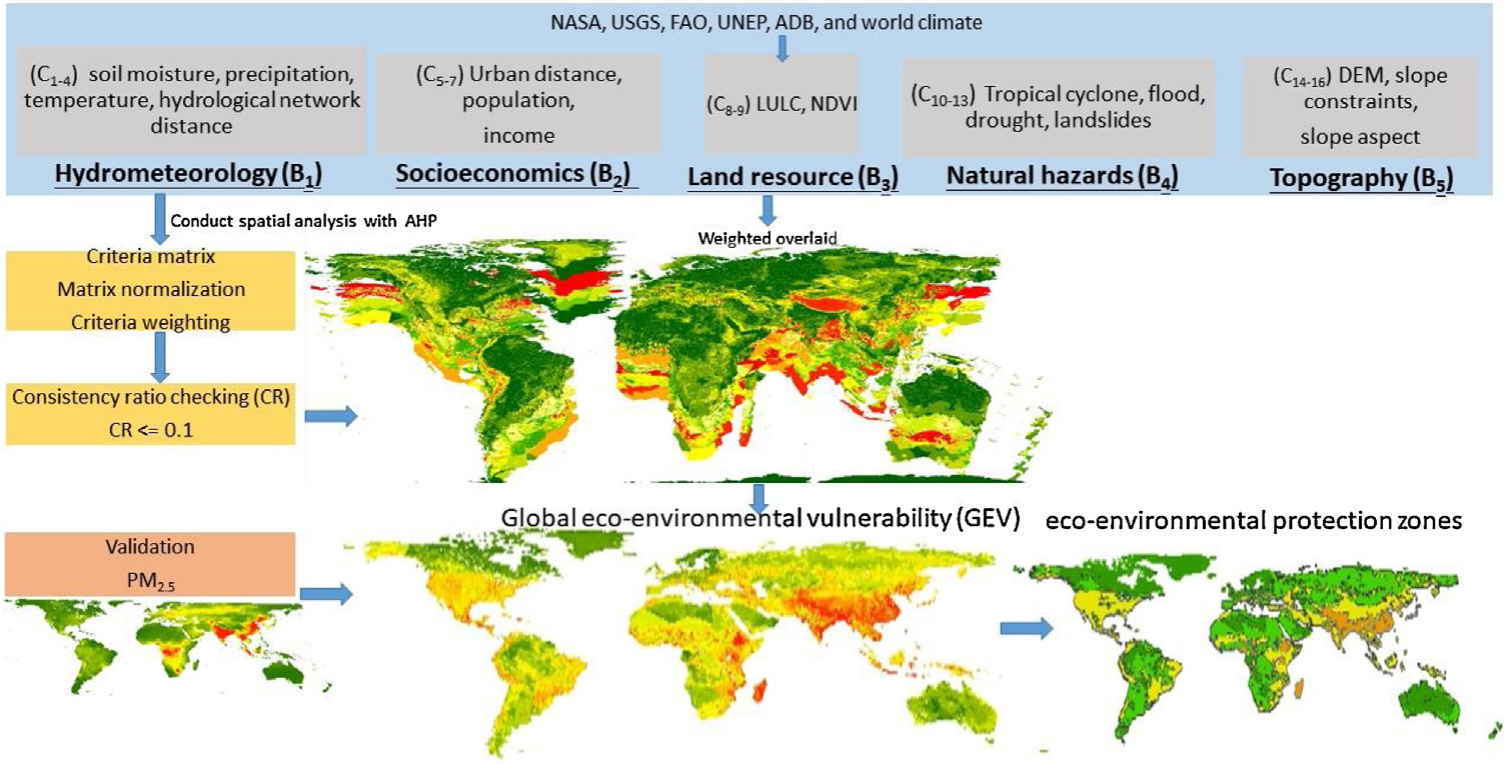
A framework for the global eco-environmental vulnerability assessment. LULC is land use/land cover; NDVI is normalized difference vegetation index; and AHP is analytical hierarchy process.

**Fig. 2:**
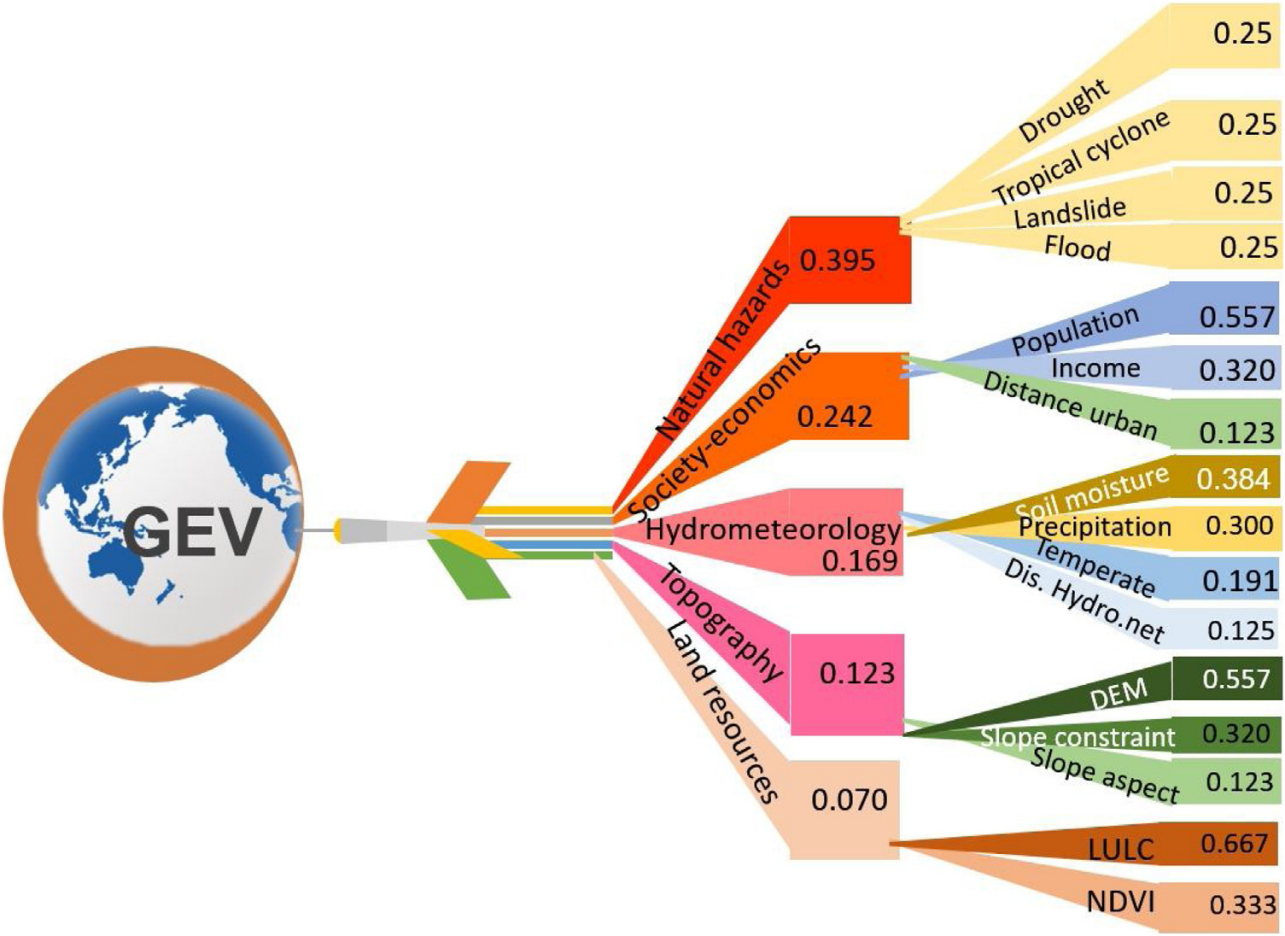
Diagram of indicators and their weights for the global eco-environmental vulnerability assessment.

The selection of influential factors and spatial data processing here are optimized to identify vulnerability levels of eco-environment at global scale due to nature and human disturbances. The influential factors can be replaced and adjusted based on the condition of the region of interest. For instance, for the region with dominated disturbance of forest fire or earthquake, the natural hazards can be replaced by the named driving forces. The global weights are derived in detail steps as given in Table 3, Table 4, Table 5, Table 6 with summary of weight results as given in Table 7. Class weights are given in Table A1 in Appendix Supplementary material. Similarly, the local weights were derived with consideration of five domains. Results of local and global weights are all summarized in diagram (Fig. 2). Data are downloaded and processed in steps as depicted in Fig. 1 and detail processing steps are described in the following:

* Step 1: Data preparation

Details of data preparation is described one by one as seen below:

**(C_1_) Soil moisture:** Annual average soil moisture is retrieved from L-band microwave missions SMOS (Soil Moisture and Ocean Salinity) as output in NETCDF format, further converted to GEOTIFF format, and then mosaicked and classified into eight classes over a global scale of 0.25-degree grid. Soil moisture is provided by INRA (Institut National de la Recherche Agronomique) and CESBIO (Centre d’Etudes Spatiales de la BIOsphère). We interpolated the areas with insufficient data or data being removed due to unsuitable values of shallow effects.

**(C_2_) Precipitation:** Global annual average precipitation (mm) with a resolution of 0.00833 ° from a GEOTIFF file is classified into eight classes. The precipitation data is interpolated using thin-plate splines with covariates including elevation, distance to the coast. Weather station data are used between 9000 and 60,000 stations with temporal range of 1970–2000 [5].

**(C_3_) Temperature:** Global average temperature with a resolution of 0.00833 ° from a GEOTIFF file is classified into eight classes. The data are processed by using the same weather station and method as precipitation data [5].

**(C_4_) Distance from hydrological network:** By using shape file formatted inland water surface data, we calculate the distances of interest by a Euclidean Distance tool in ArcGIS, which are then further classified into eight classes.

**(C_5_) Population:** Population data are in excel file format, stored into a shape file, and further converted to GEOTIFF format. Finally, population is classified into eight classes.

**(C_6_) Income:** Income data are in excel file format, stored into a shape file, and then further converted to GEOTIFF format. Raster of income is classified into five classes.

**(C_7_) Distance from urbanized areas:** Urban areas are in shape file format. We calculate the distance by a Euclidean distance tool in ArcGIS, which is further classified into eight classes from near range to far range from the urbanized areas.

**(C_8_) Land use/land cover (LULC):** The MODIS Land Cover Type product in 2017 (Short Name: MCD12Q1) of 500 m SIN Grid in HDF format is download and further processed such as mosaicked and converted into GEOTIFF format and then classified into 16 classes following the legend and instruction of providers. References include USGS’s website and following papers [[18], [19]].

**(C_9_) Normalized Difference Vegetation Index (NDVI):** Global MODIS vegetation indices, NDVI product Vegetation Indices 16-Day L3 Global 500 m are downloaded, mosaicked, and used to compute mean value NDVI of the year 2017, and then further classified into five classes.

**(C_10_) Drought; (C_11_) Tropical cyclones; (C_12_) Landslides; (C_13_) Flood:** These indicators are downloaded either in an excel file, shape file or raster GEOTIFF format, then further processed into same format of GEOTIFF at resolution of 0.0833 ° over a global scale, and aggregated by weighted sum function in ArcGIS (each type of natural hazard has the same weight). Final raster of natural hazards is classified into five classes.

**(C_14_) DEM:** SRTM DEM in GEOTIFF file is downloaded with a resolution of 0.00833 ° and further processing is conducted such as filling holes, and classification into eight classes.

**(C_15_) Slope constraint:** Slope constraint with 0.0833 ° resolution is downloaded and converted into GEOTIFF file and classified into 8 classes from low constraint to very frequent constraint.

**(C_16_) Slope aspect:** Slope aspects are computed by using ArcGIS function with input SRTM DEM and then further classified into 10 classes.

**PM_2.5 -_ validation data:** An annual global map of PM_2.5_ is derived from MODIS data [[20], [21]].

*Step 2: Derivation of global weights

Analytical Hierarchy Process (AHP)

AHP is an effective tool for dealing with a larger number of influential variables for making decisions in a structural way. Take for example risk, hazard, and eco-environmental impact in a multi-indicator decision making process to derive vulnerability assessment [[22], [23]], AHP offers the analysis of the spatial multi-indicator layers through the creation of a hierarchical structure by providing weighting and ranking with the views of expert opinions and users [[23], [24]]. Here a weighted overlay technique is used to synthesize weighted and ranked spatial variables together. AHP has been widely used for mapping hazards, vulnerability, and risk of various natural disasters such as floods, landslides. Hence, it is considered suitable for global eco-environmental vulnerability assessment [[25], [26]]. In this study, for weighting the selected indicators of eco-environmental vulnerability, the individual pairwise comparison matrix was established through the qualitative judgments of five experts, and a user, each of whom considered the influential factors and alternatives, which were integrated with every indicator. For prioritization of the indicators, experts were asked based on the pairwise comparison 9 point scales (Table 2) developed by Saaty (2008) [22]. Five experts were selected at the international and national levels. The expert selection was based on their related depth knowledge and research experiences about the influential factors. The selected experts were from research institutions, and governmental and academic sectors.

**Table 2:** Scale of relative importance (adapted from Saaty, 2008).

Relative importance	Definition	Description
1	Equal importance	Two indicators influence on objective equally
3	Moderate importance	Experience and judgement slightly favor one indicator over another
5	Strong importance	Experience and judgement strongly favor one indicator over another
7	Very strong importance	One decision indicator is favored strongly over another and its supremacy is established in practice
9	Extreme importance	The evidence favoring one decision indicator over another is of the highest possible order of validity
2, 4, 6, 8	Intermediate values between the two adjacent judgements	Compromise is needed

**Table 3:** Pairwise comparison of group variables.

Group variables	Natural hazards	Social economics	Topography	Hydrometeorology	Land resources
Natural hazards	1	3	2	3	4
Social economics	1/3	1	3	2	3
Topography	1/2	1/3	1	1	2
Hydrometeorology	1/3	1/2	2	1/2	3
Land resources	1/4	1/3	1/2	1/3	1

**Table 4:** Pairwise comparison matrix of group variables to derive global weights.

Group variables	Natural hazards	Social economics	Topography	Hydrometeorology	Land resources	5th Root of index	Priority vector
Natural hazards	1.000	3.000	2.000	3.000	4.000	2.352	0.377
Social economics	0.333	1.000	3.000	2.000	3.000	1.431	0.229
Topography	0.500	0.333	1.000	0.500	2.000	0.699	0.112
Hydrometeorology	0.333	0.500	2.000	1.000	3.000	1.000	0.160
Land resources	0.250	0.333	0.500	0.333	1.000	0.425	0.068
Column Sum	2.417	5.167	8.500	6.833	13.000	6.236	1.000
Priority row	0.912	1.186	0.953	1.096	0.886

**Table 5:** Normalized pairwise comparison matrix, weights, and consistency ratio (CR).

Group variables	Natural hazards	Society- economic	Topography	Hydrological network	Land resources	Weight (Average)	Row totals	Row totals/ average
Natural hazards	0.414	0.581	0.235	0.439	0.308	0.395	2.157	5.456005
Social economics	0.138	0.194	0.353	0.293	0.231	0.242	1.293	5.353721
Topography	0.207	0.065	0.118	0.073	0.154	0.123	0.627	5.089405
Hydrometeorology	0.138	0.097	0.235	0.146	0.231	0.169	0.880	5.193529
Land resources	0.103	0.065	0.059	0.049	0.077	0.070	0.368	5.218952
Column Sum	1.000	1.000	1.000	1.000	1.000	1.000	5.325

**Table 6:** Checking consistency of judgments.

Checking methods	Geometric mean method	Row average method
λmax	5.032	5.262
Consistency index (CI)	0.008	0.066
n (number of variables)	5	5
Random index (RI)	1.110	1.110
Consistency ratio (CR)	0.007	0.059

**Table 7:** Weightings of group indicators and indicators used for the calculation of global eco-environmental vulnerability (modified and adapted from (1,15). Consistency ratio of assessment is 0.007. Class weights and consistency ration of each indicator are provided in Table 6.

Group variables/ Factors (*B_i_*)	Global weight (*W_i_*)	Variables/Factors (*C_j_*)	Local weight (*w_j_*)
B_1_. Hydrometeorology	0.169	C_1_ Soil moisture	0.384
		C_2_ Precipitation	0.300
		C_3_ Temperate	0.191
		C_4_ Distances from hydrological network	0.125
B_2_. Society-economics	0.242	C_5_ Population	0.557
		C_6_ Income	0.320
		C_7_ Distances from urbanized areas	0.123
B_3_. Land resources	0.070	C_8_ LULC	0.667
		C_9_ NDVI	0.333
B_4_. Natural hazards	0.395	C_10_ Drought	0.250 0.250 0.250 0.250
		C_11_ Tropical cyclone	
		C_12_ Landslide	
		C_13_ Flood	
B_5_. Topography	0.123	C_14_ DEM	0.557
		C_15_ Slope constraint	0.320
		C_16_ Slope aspect	0.123

The consistency ration (CR) was calculated to justify the consistency of comparisons given by experts and the user in the pairwise comparison matrix. The comparisons in the pairwise comparison matrix are considered consistent if the CR is equal or less than 0.1. The CR is calculated using following equation:

(1) CR = Consistency Index/Random Index

where Random Index (RI) represents the randomly generated average consistency index (CI), which is defined as follows:

(2) $$CI=(\lambda_{\max}-n)/(n-1)$$

where $\lambda_{\max}$ refers to the largest eigenvalue of the matrix and n represents the order of the matrix (1).

In addition, the Natural Break Statistical method in ArcGIS environment was used during the spatial analysis to classify the indicator layers where it was required. All the classification of indicator layers and internal weights are explained in Table 7.

## Derivation of global weights

For determining the weights of the global group variables, the individually pairwise comparison matrix was established by using the 9-point scale (Table 2) of Saaty (2008) as shown in Table 3 that is further processed to obtain the pairwise comparison matrix of group variables as shown in Table 3. The table of normalized pairwise comparison matrix, weights, and consistency ratio (CR) is then given in Table 5 followed by Table 6 showing the consistency of judgments. Two types of consistency methods, including geometric mean method and row average method, are used to check the consistency of our judgments, requiring the consistency ratio to be less than 0.1. From Table 6, it is seen that our judgments are acceptable. Under such circumstance, the derived global weights are adopted for further analysis.

*Step 3: Classification of indicators and derivation of class weights

Similarly, the class weights are derived as shown in Table 7.

*Step 4: Mapping four major determinants and final global eco-environmental vulnerability maps

**Fig. 3:**
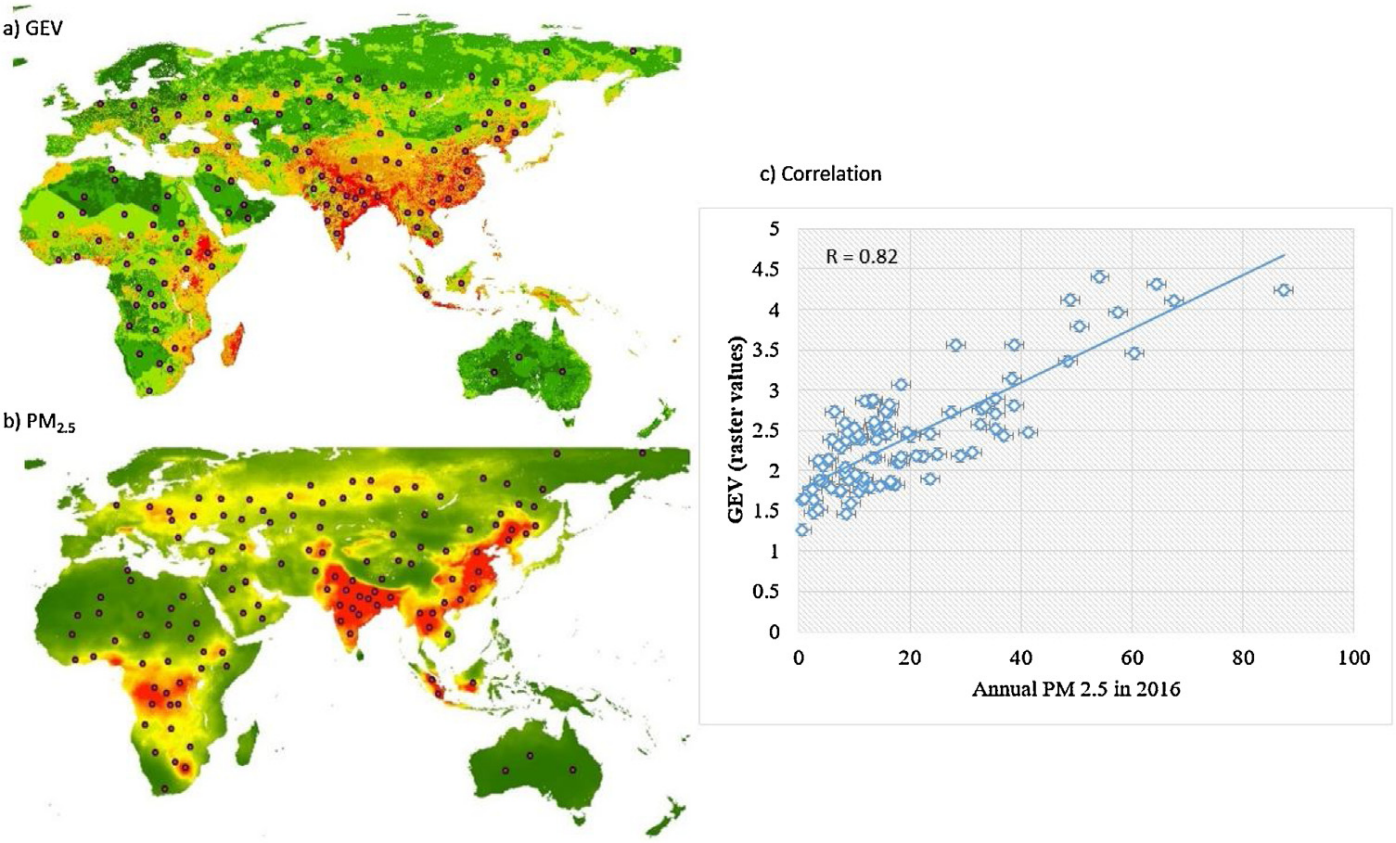
Comparison of (a) the global eco-environmental vulnerability map with (b) annual PM2.5 distribution in 2016. (c) Correlation coefficient between (a) and (b) is 0.82 for 100 randomly chosen checking points over the globe.

**Fig. 4:**
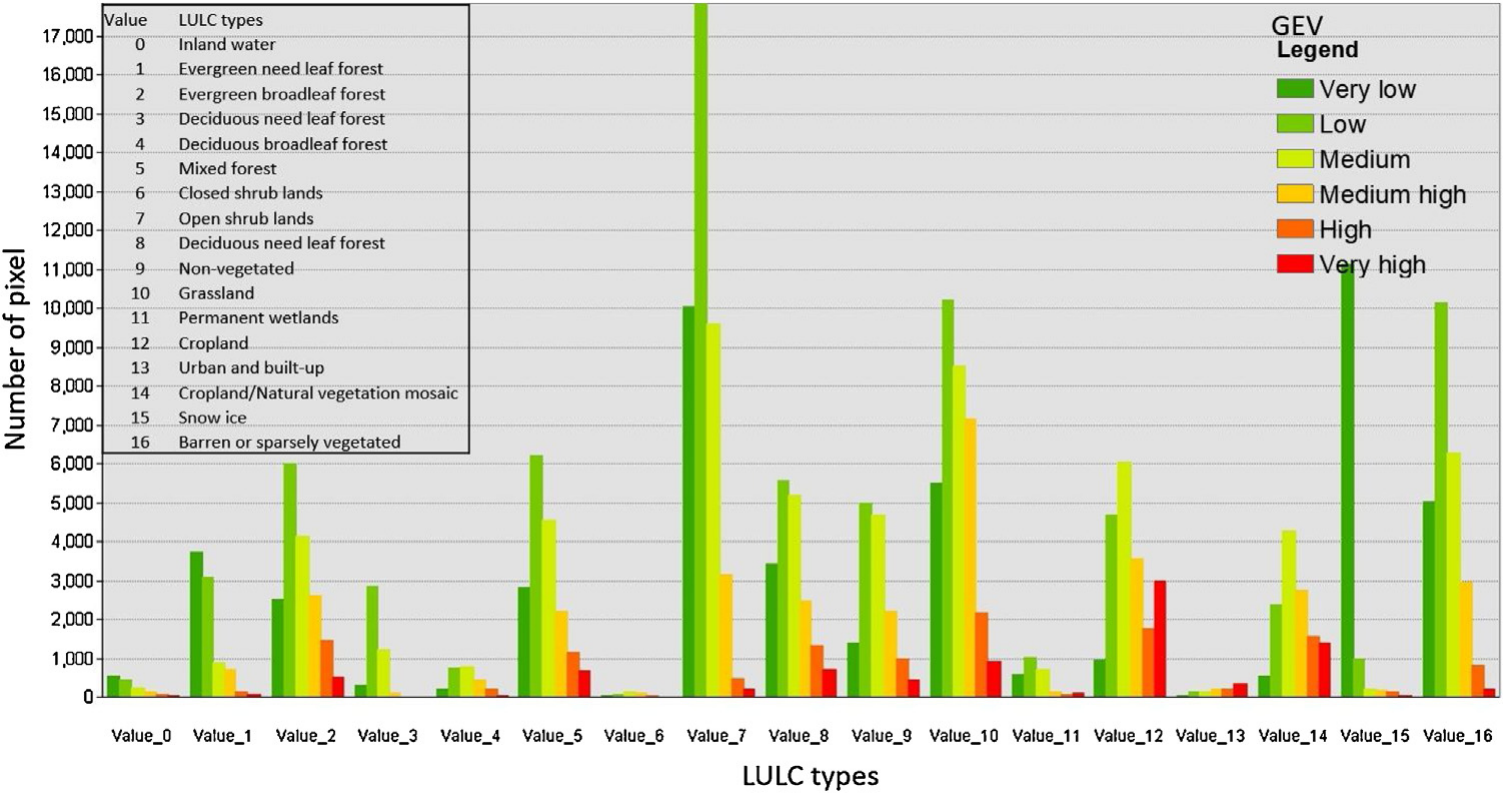
Distribution of eco-environmental vulnerability with LULC.

**Fig. 5:**
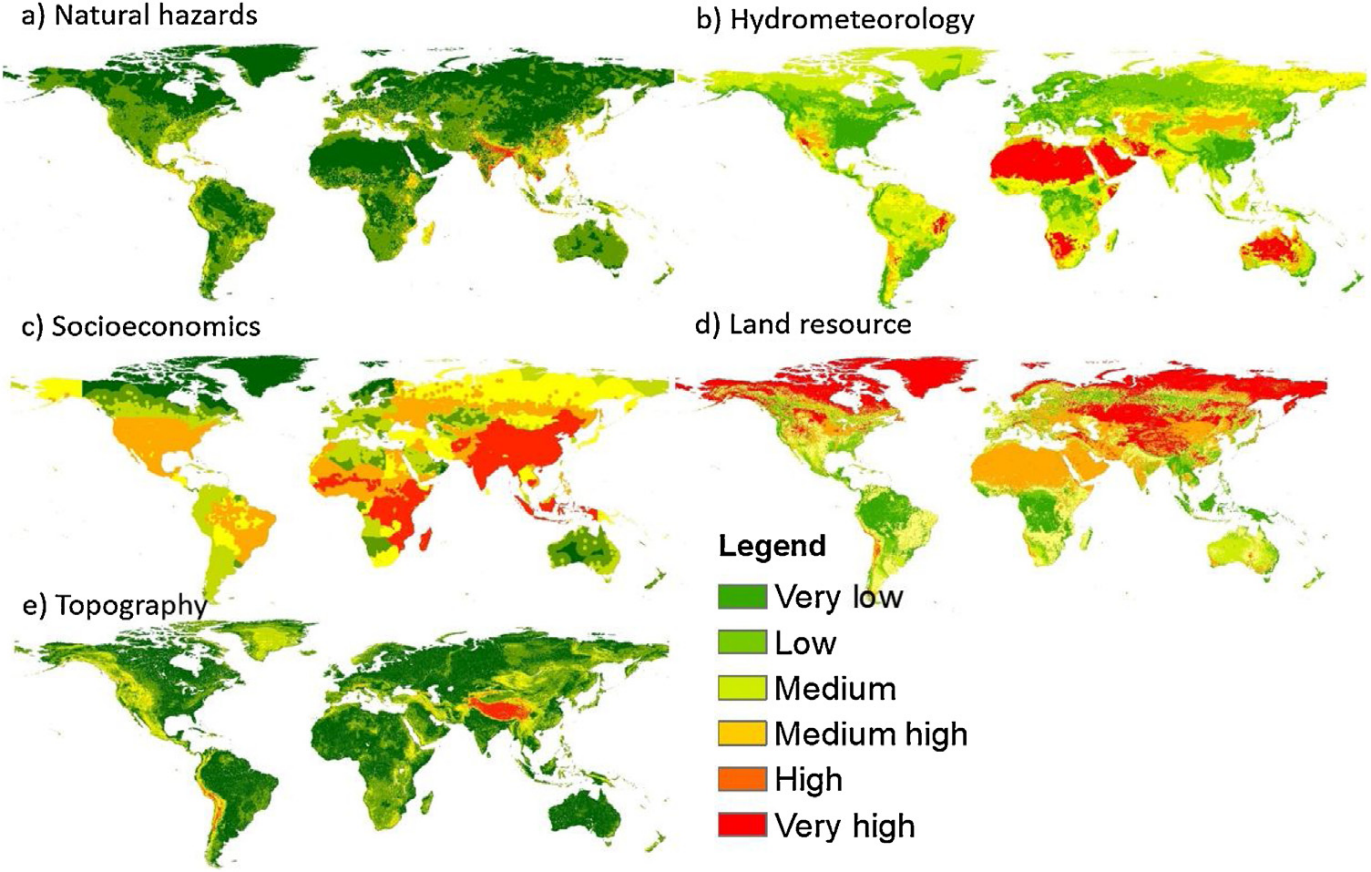
Five major disturbance determinants of global eco-environmental vulnerability: (a) Natural hazards; (b) Hydrometeorology; (c) Socioeconomics; (d) Land resource; and (e) Topography.

**Fig. 6:**
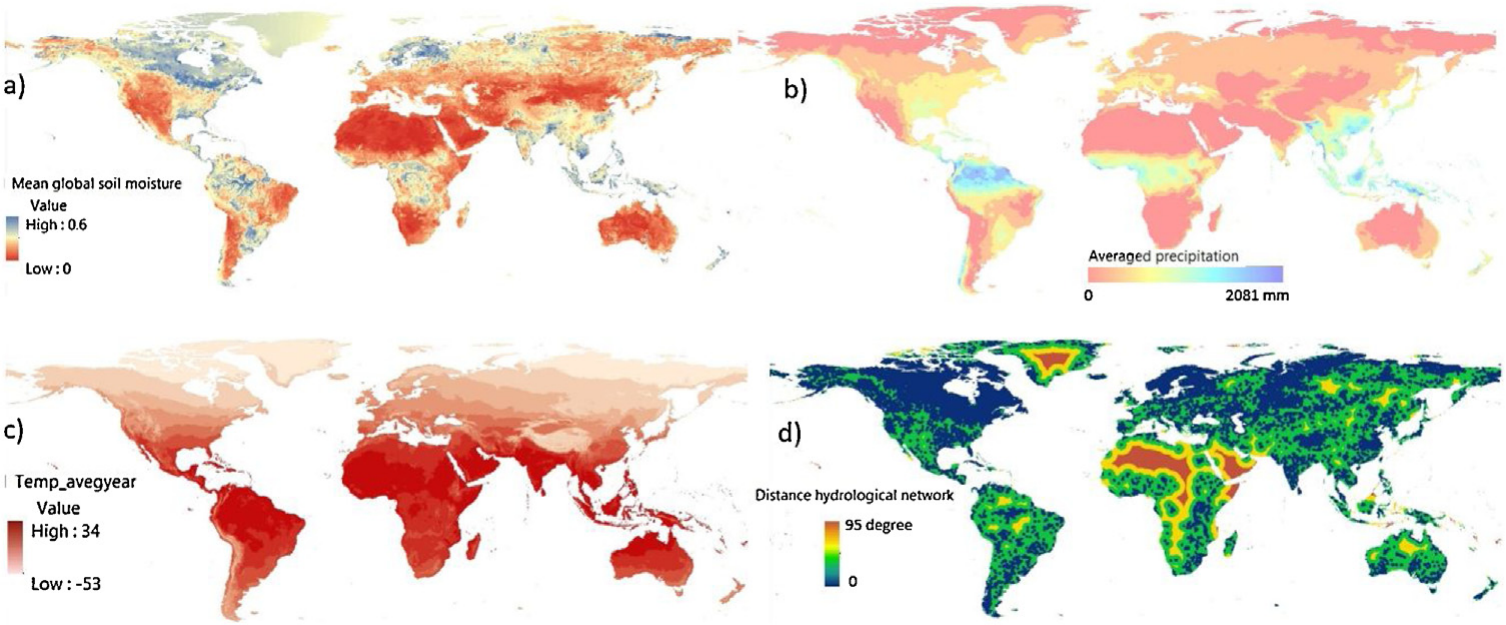
Indicators of hydrometeorology include (a) mean soil moisture, (b) precipitation, (c) temperature, and (d) distance from hydrological network.

**Fig. 7:**
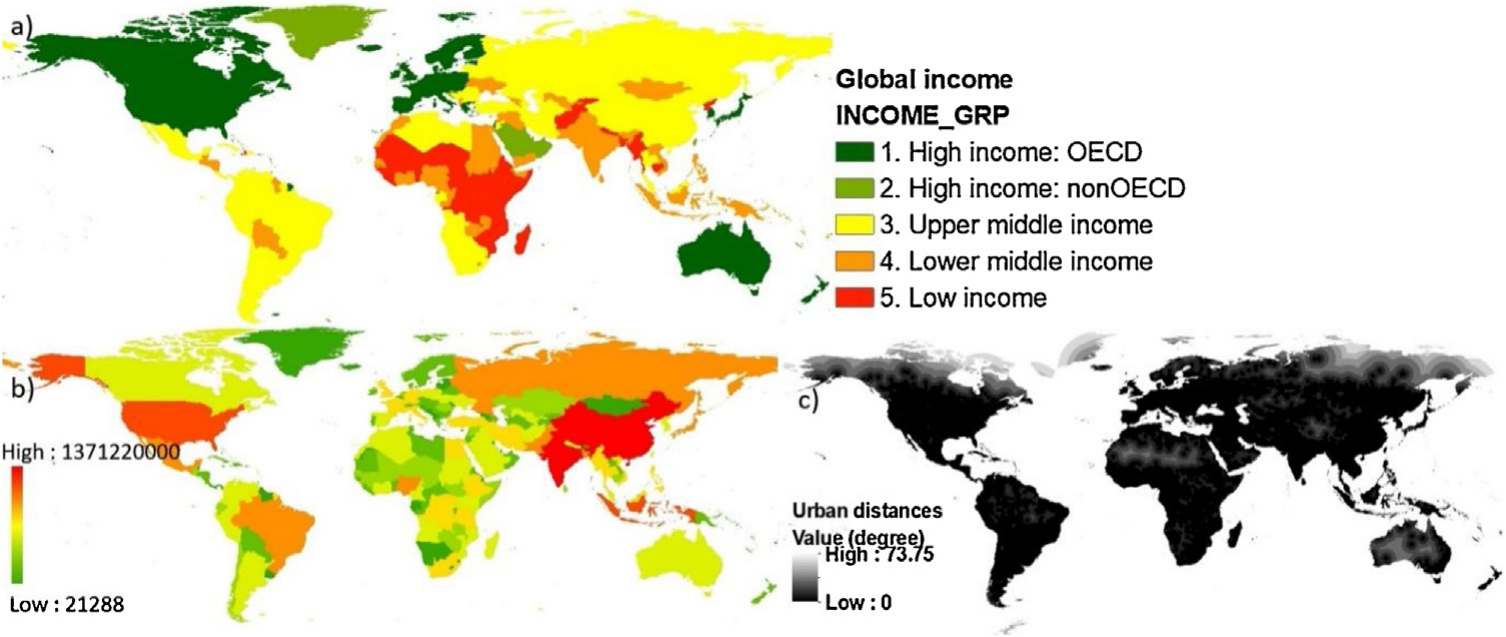
Indicators of socioeconomics: (a) income; (b) population; and (c) distance from urbanized areas.

An analytical hierarchy process (AHP) and geographical information system (GIS) are implemented to combine multi-indicators in groups and then further aggregated to become one final indicator of GEV by using Eqs. (3) and (4):

(3) $$B_{i}=\sum_{1}^{nBi}C_{j}*w_{j}$$

(4) $$GEV=\sum_{1}^{5}B_{i}*W_{i}$$

where GEV denotes the global eco-environmental vulnerability (the higher the GEV value, the greater the vulnerability is likely to be), B_i_ is the ith group determinant factor, W_i_ is the weight of the ith group determinant factor, CJ is the jth indicator, wj is the weight of the jth variable, and nB_i_ is the number of indicators in a group determinant factor B_i_ introduced in Table 1 and Step 1 data preparation. Weights of 16 indicators and five groups are presented in the Table 7 and Fig. 3, Fig. 4, Fig. 5, Fig. 6, Fig. 7. To classify vulnerable intensity, the GEV is standardized and compared. In this study, we use histograms to reveal the statistical distribution corresponding to values of grid cells of eco-environmental vulnerability raster to classify GEV assessment into six categories, namely very low (< 1.66), low (1.66–2.13), medium (2.13–2.56), medium high (2.56–3.06), high (3.06–3.59), and very high (>3.59).

*Step 5: Analysis of the result and validation

**Fig. 8:**
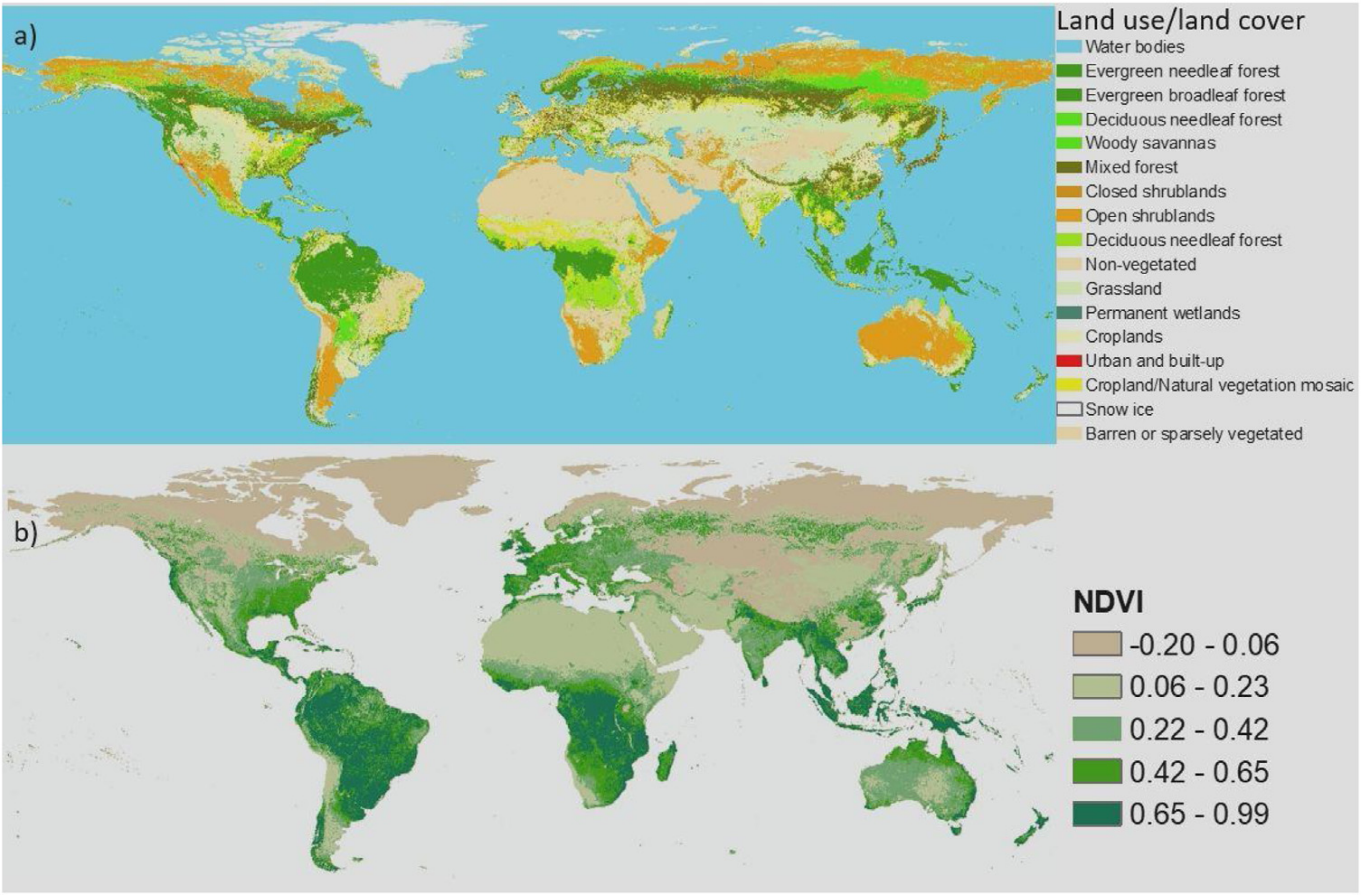
Indicators of land resources: (a) LULC and (b) NDVI.

**Fig. 9:**
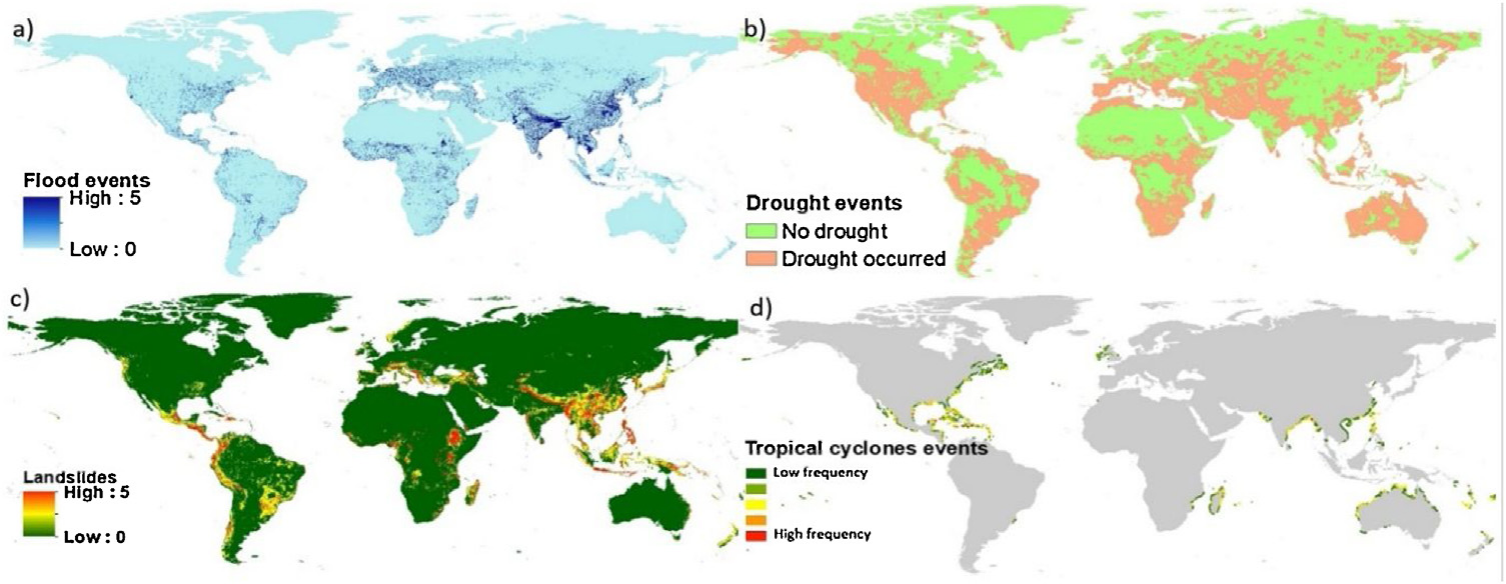
Indicators of natural hazards: (a) flood, (b) drought, (c) landslide, and (d) tropical cyclone frequency.

**Fig. 10:**
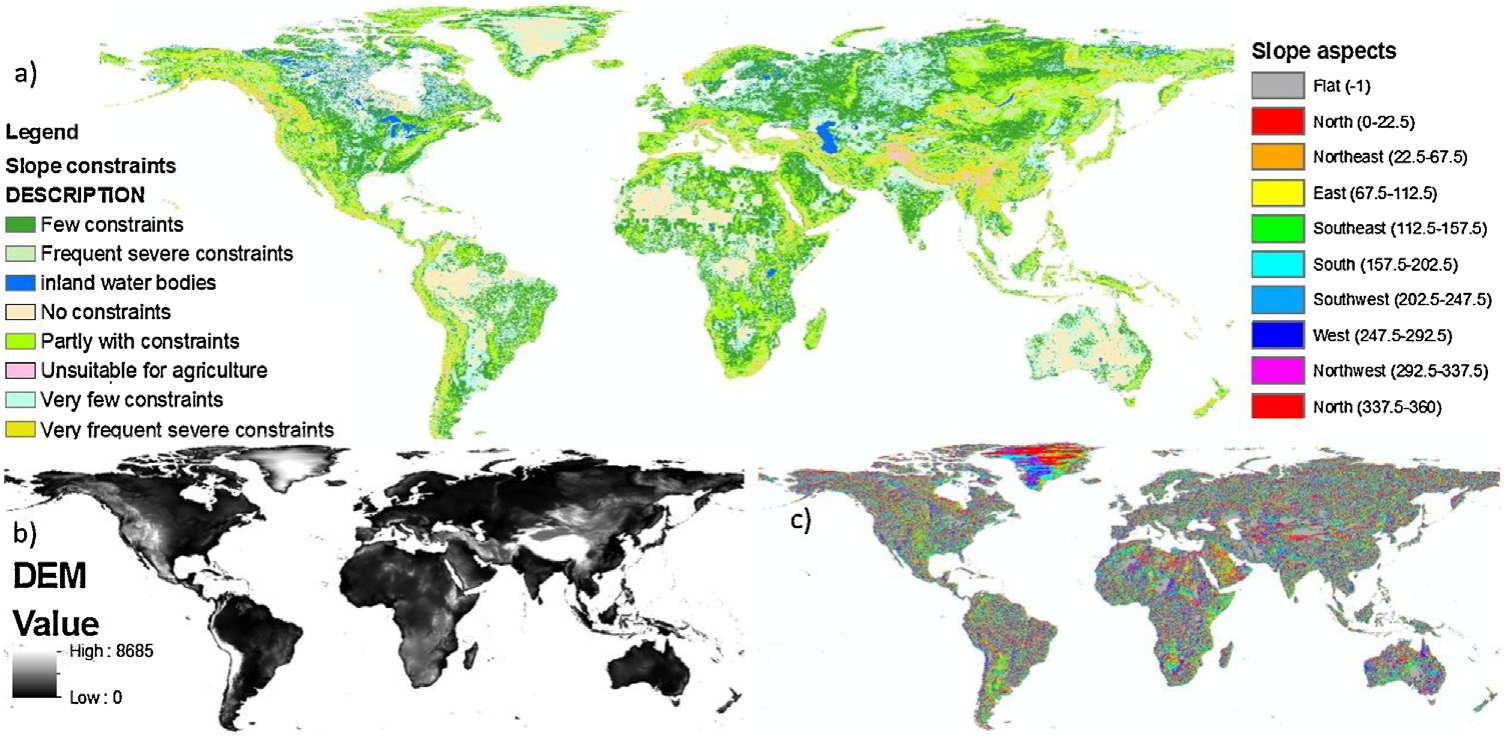
Indicators of topography: (a) slope constraint, (b) DEM, and (c) slope aspect.

After obtaining the maps of four major influential factors (Fig. 5) and global eco-environmental vulnerability map, the spatial analysis distribution is necessary to understand how it exhibits across to continents with influential factors. Spatial Analysis Tools including, Zonal Histogram, Tabulate Area and Zonal Statistic in ArcGIS are applied to investigate the statistical features of these maps. For example, Tabulate Area distribution of eco-environmental vulnerability over the land cover types is shown in Fig. 4. Individual indicators are presented in Fig. 6, Fig. 7, Fig. 8, Fig. 9, Fig. 10.

Validation is crucial to check the reliability of the results. A global map of PM_2.5_ is derived from MODIS data [21] and considered as an independent variable for validation with global eco-environmental vulnerability map since it can be considered as an anthropogenic disturbance associated with nature and human-made influence. We choose 100 points randomly distributed over the globe by using the Random Points and Pixel Value to Points functions to get the attributing values of global eco-environmental raster and PM_2.5_ raster. Analysis of correlations between EVA map results and PM_2.5_ pollution distribution is conducted. It is found that the correlation coefficient reaches 0.82 as shown in Fig. 3.
