# Supplementary material for: Mapping global eco-environment vulnerability due to human and nature disturbances
Source: MethodsX. 2019 Apr 11;6:862–75. doi: 10.1016/j.mex.2019.03.023 (PMC6495093; doi:10.1016/j.mex.2019.03.023)
Supplement: Supplementary file 1 [file mmc1.docx]

**Appendix**

**Table A1.** Class weights and consistency ratio of indicator layers.

| **Components of disturbances** | **Indicators** | **Classes** | **Class weights** |
| --- | --- | --- | --- |
| **Hydrometeorology (*B_1_*)** | **Soil moisture (%)**  **(*C_1_*)**  Consistency Ratio (CR) = 0.035 | (1) 0.00 - 0.06  (2) 0.06 - 0.11  (3) 0.11 - 0.15  (4) 0.15 - 0.20  (5) 0.20 - 0.25  (6) 0.25 - 0.31  (7) 0.31 - 0.39  (8) 0.39 - 0.59 | 0.377  0.145  0.069  0.047  0.032  0.023  0.101  0.206 |
|  | **Precipitation (mm)**  **(*C_2_*)**  CR = 0.035 | (1) 0 - 25  (2) 25 - 65  (3) 65 - 122  (4) 122 - 188  (5) 188 - 261  (6) 261 - 334  (7) 334 - 440  (8) 440 – 2018 | 0.206  0.101  0.047  0.023  0.032  0.069  0.145  0.377 |
|  | **Temperature ( ^o^ C degree)**  **(*C_3_*)**  CR = 0.035 | (1) < -10  (2) -10 - 0  (3) 0 - 5  (4) 5 - 10  (5) 10 - 15  (6) 15 - 20  (7) 20 -25  (8) 25 – 34 | 0.206  0.101  0.023  0.032  0.047  0.069  0.145  0.377 |
|  | **Distance from hydrological network (degree)**  **(*C_4_*)**  CR = 0.035 | (1) 0 – 074  (2) 0.74 – 2.609  (3) 2.609 – 5.212  (4) 5.212 – 18.26  (5) 18.26 – 36.16  (6) 36.16 – 59.64  (7) 59.64 – 73.06  (8) 73.06 – 95.06 | 0.023  0.032  0.047  0.069  0.101  0.145  0.206  0.377 |
| **Socioeconomics (*B_2_*)** | **Population (people)**  **(*C_5_*)**  CR = 0.035 | (1) 21,288 – 3,9699,249  (2) 3,969,249 – 11, 629, 553  (3) 11,629,553 – 20,966,000  (4) 20,966,000 – 51, 014,947  (5) 51,014,947 – 101,716,359  (6) 101,716,359 – 205,962, 108  (7) 205,962,108 – 320,896,618  (8) 320,896,618 – 1, 371,220,000 | 0.377  0.206  0.145  0.101  0.069  0.047  0.032  0.023 |
|  | **Income** (five categories based on world bank standard)  **(*C_6_*)**  CR = 0.031 | (1) High income OECD  (2) High income non-OECD  (3) Upper middle income  (4) Lower middle income  (5) Low income | 0.074  0.050  0.120  0.256  0.500 |
|  | **Distance from urban** (degree)  **(*C_7_*)**  CR = 0.035 | (1) 0 – 4.6  (2) 4.6 – 11.6  (3) 11.6 – 19.1  (4) 19.1 – 27.2  (5) 27.2 – 36.1  (6) 36.1 - 46  (7) 46 - 56  (8) 56 – 73.8 | 0.377  0.206  0.145  0.101  0.069  0.047  0.032  0.023 |
| **Land resource (*B_3_*)** | **Land use/land cover**  **(LULC)**  **(*C_8_*)**  CR = 0.05 | (1) Inland water  (2) Evergreen needleaf forest  (3) Evergreen broadleaf forest  (4) Deciduous needleaf forest  (5) Deciduous broadleaf forest  (6) Mixed forest  (7) Closed shrub land  (8) Open shrub lands  (9) Deciduous needleaf forest  (10) Non-vegetated  (11) Grassland  (12) Permanent wetlands  (13) Cropland  (14) Urban and built-up  (15) Cropland/Natural vegetation mosaic  (16) Snow ice  (17) Barren or sparsely vegetated | 0.013  0.019  0.022  0.031  0.038  0.05  0.054  0.059  0.031  0.118  0.105  0.085  0.136  0.198  0.136  0.073  0.118 |
|  | **Normalized Difference Vegetation Index (NDVI)**  **(*C_9_*)**  CR = 0.030 | (1) -0.20 – 0.06  (2) 0.06 – 0.23  (3) 0.23 – 0.42  (4) 0.42 – 0.65  (5) 0.65 – 099 | 0.440  0.233  0.139  0.106  0.082 |
| **Natural hazards (*B_4_*)** | **Drought (*C_10_*)** | All of these natural hazard indicators are equally integrated and classified into six classes of vulnerability directly and have same weight | 0.250 |
|  | **Tropical cyclones (*C_11_*)** |  |  |
|  | **Landslides (*C_12_*)** |  |  |
|  | **Flood**  **(*C_13_*)** |  |  |
| **Topography (*B5*)** | **DEM** (meter)  **(*C_14_*)**  CR = 0.035 | (1) 0 - 259  (2) 259 - 585  (3) 585 - 1,001  (4) 1,001 - 1,537  (5) 1.537 - 2,237  (6) 2,237 – 3,231  (7) 3,231 – 4,378  (8) 4,378 – 8,685 | 0.023  0.032  0.047  0.069  0.101  0.145  0.206  0.377 |
|  | **Slope constraint**  **(*C_15_*)**  CR = 0.035 | (1) Inland water bodies  (2) No constraints  (3) Very few constraints  (4) Few constraints  (5) Partly with constraints  (6) Frequently severe constraints  (7) Very frequently severe constraints  (8) Unsuitable for agriculture | 0.023  0.032  0.047  0.069  0.101  0.145  0.206  0.377 |
|  | **Slope aspect** (degree)  **(*C_16_*)**  CR = 0.008 | (1) Flat (−1)  (2) North (0–22.5)  (3) Northeast (22.5–67.5)  (4) East (67.5–112.5)  (5) Southeast (112.5–157.5)  (6) South (157.5–202.5)  (7) Southwest (202.5–247.5)  (8) West (247.5–292.5)  (9) Northwest (292.5–337.5)  (10) North (337.5-360) | 0.026  0.071  0.189  0.071  0.071  0.071  0.071  0.345  0.071  0.071 |
