# Supplementary material for: Mapping global eco-environment vulnerability due to human and nature disturbances
Source: MethodsX. 2019 Apr 11;6:862–75. doi: 10.1016/j.mex.2019.03.023 (PMC6495093; doi:10.1016/j.mex.2019.03.023)
Supplement: Supplementary file 2 [file mmc2.pdf]

Science of the Total Environment 664 (2019) 995–1004

Contents lists available at ScienceDirect

Science of the Total Environment

journal homepage: www.elsevier.com/locate/scitotenv

Global mapping of eco-environmental vulnerability from human and nature disturbances

Kim-Anh Nguyen [a,b,c], Yuei-An Liou [a,b,*]

[a] Center for Space and Remote Sensing Research, National Central University, 300, Jhongda Rd., Jhongli District, Taoyuan City 32001, Taiwan, ROC
[b] Taiwan Group on Earth Observations, Hsinchu, Taiwan, ROC
[c] Institute of Geography, Vietnam Academy of Science and Technology, 18 Hoang Quoc Viet Rd., Cau Giay, Hanoi, Viet Nam

HIGHLIGHTS

- A global eco-environmental vulnerability map is generated by using a proposed assessment framework and essential datasets.
- Significant eco-environmental vulnerability levels are widely attributed in Asia and Africa (China, India and Ethiopia).
- Natural hazards and anthropogenic stress pose a threat on eco-environment and enhanced by climate change.
- Advised eco-environmental protection zones provide critical information for environmental management and conservation.

GRAPHICAL ABSTRACT

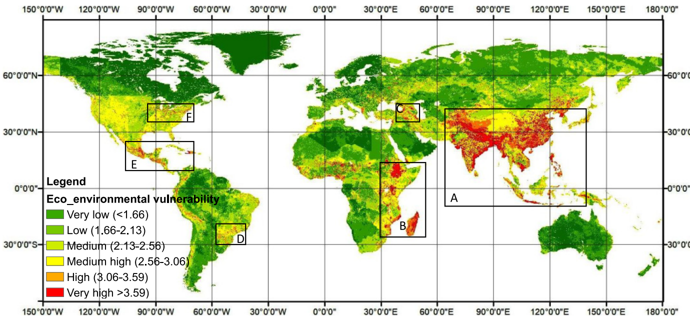

A Global eco-environmental vulnerability map.

ARTICLE INFO

Article history:
Received 8 October 2018
Received in revised form 27 January 2019
Accepted 30 January 2019
Available online 05 February 2019

Editor: Daniel Wunderlin

Keywords:
Eco-environmental vulnerability
Natural and human-induced disturbances
Analytical hierarchy process (AHP)
Geographical information system (GIS)

ABSTRACT

Global environments are threatened by intensively natural variation and continuously increased human-made disturbances. Assessment of the global eco-environment vulnerability (global EV or GEV) caused by both natural and human-induced disturbances plays a key role in providing valuable information about ecological and environmental background for designing suitable policy measures to improve and restore environment. We present the first global-scale map of quantified eco-environmental vulnerability by integrating remote sensing, GIS modelling, and global census datasets, employing 16 influential factors across five domains: *socioeconomics, land resources, natural hazards, hydrometeorology, and topography*. The GEV is classified into six levels, namely *very low vulnerability, low vulnerability, medium vulnerability, medium high vulnerability, high vulnerability*, and *very high vulnerability*. At global scale, a small fraction of the globe (10.1%) is strongly (*high* and *very high vulnerability*) affected by influential factors. Among continents, the largest fraction of *very high vulnerability* level is attributed to Asia (74.6%) followed by Africa (19.6%). National-scale analysis shows that China and India are the most vulnerable in Asia and in the world. Our study provides accumulative impacts of manmade and natural disturbances, which are vital for decision makers to set improvement targets on specific areas over local, regional, and global scales, and design and adopt new practices to lessen natural and manmade disturbances on environment, while keeping track of evolution of the other environmental aspects.

© 2019 Published by Elsevier B.V.

* Corresponding author at: Center for Space and Remote Sensing Research, National Central University, 300, Jhongda Rd., Jhongli District, Taoyuan City 32001, Taiwan, ROC.
E-mail address: yueian@csrsr.ncu.edu.tw (Y.-A. Liou).

https://doi.org/10.1016/j.scitotenv.2019.01.407
0048-9697/© 2019 Published by Elsevier B.V.

1. Introduction

Human activities and natural variations affect the eco-environment through direct and indirect means (Savo et al., 2016; Stern, 1993; Rosa and Dietz, 1998; Lambin et al., 2001; Ippolito et al., 2010). Understanding, quantifying, and mapping the spatial distribution of vulnerable magnitude levels of eco-environment caused by manmade and natural impacts are needed for eco-environmental protection and restoration (Webster et al., 2005; Janssen and Ostrom, 2006; Garnier et al., 2017). Conventionally, the eco-environmental vulnerability or traditional vulnerability is generally considered as a component of exposure, sensitivity, and adaptive capacity under concerns of physical, economic, social, environmental, and cultural aspects (Turner et al., 2003; Adger, 2006; Raúl et al., 2017). Given the scope of this study, the term "*eco-environmental vulnerability*" (EV) is defined as the risk of damage to the natural environment or particular ecosystem because of any disturbances, including internal physical/structural features and external dynamics (Nguyen et al., 2016). Numerous studies about eco-environmental vulnerabilities at different spaces, timespans, and aspects were conducted (Eitner, 2016; Aretano et al., 2017; Fawcett et al., 2017; Rajesh et al., 2018; Raufirad et al., 2018). A vast amount of spatiotemporally distributed global datasets become easier accessed with advanced science and technology in remote sensing, mapping, data analysis efforts, and environmental or weather models. Nevertheless, the use of global datasets in previous studies related to eco-environmental vulnerability evaluation was mainly applied or limited to regional, provincial, and national scales (Jongman et al., 2015; Liou et al., 2017; Brooks, 2003). In addition, conceptual frameworks for assessing eco-environmental vulnerability were introduced in various ways in the literature (Adger et al., 2013; Eakin and Luers, 2006; De Lange et al., 2010; Hinkel, 2011; Berrouet et al., 2018). These research outcomes have certainly provided powerful tools for the design of policy instruments for environmental conservation and management at regional levels. However, they rarely provided global inland spatial visualization of eco-environmental vulnerability except, as far as we know, a study about human impacts on the global marine ecosystems conducted by Halpern et al. (2008), which presented an overall profile of human impacts on the world's oceans. Note that it is infeasible to assess global eco-environmental vulnerability by synthesizing national-regional scale mappings with inconsistent methods and definitions.

Currently, there exist different ways to achieve eco-environmental evaluation. For example, one of them is addressed by looking at natural drivers, such as climate changes through examination of precipitation, temperature, evapotranspiration, and sea level rise (Janssen and Ostrom, 2006; Ericson et al., 2006; Metzger et al., 2005). The second way is to investigate eco-environmental vulnerability by considering human and social dynamics (Ippolito et al., 2010; Johnson et al., 2016; Kumar et al., 2016; Viles and Cutler, 2012). However, various underlying factors are brought up due to complexity in interactions between human and nature, which significantly affect the environment. So far, there have been no general rules for selecting how many variables are required to evaluate the eco-environmental vulnerability. Accordingly, it is necessary to develop a reliable way to comprehensively characterize the response of eco-environment on both natural and human driving forces. In this study, we use global datasets to develop an eco-environmental vulnerability map over a global scale by synthesizing the accumulative impacts of five dimensions including *hydrometeorology, natural hazards, socioeconomics, topography,* and *land resources*. Such a global environmental vulnerability map is the first time presented in the literature as far as we know. It identifies the global hotspots for environmental researchers and decision makers around the world to take further measures for the purposes of sustainability, conservation, and development. We captured the five dimensions via 16 selective indicators: 1) soil moisture; 2) precipitation; 3) temperature; 4) distance from hydrological network; 5) population; 6) income; 7) distance from urbanized areas; 8) land use/land cover; 9) Normalized Difference Vegetation Index (NDVI); 10) drought; 11) tropical cyclones; 12) landslides; 13) flood; 14) DEM; 15) slope constraint; and 16) slope aspect. Subsequently, three eco-environmental zones are introduced with functions and advices for activities and planning for the regions of concern. Concerning both natural and manmade disturbances, global-scale eco-environmental vulnerability analysis can be of value in (*i*) Enriching the guidance of global and regional planning and construction, and protection of the ecological environment; (*ii*) Harmonizing information from the reports that employ different approaches or definitions; (*iii*) Providing a feasible framework template of eco-environmental vulnerability assessment, which benefits environmental education; and (*iv*) Conveying information to the public for enhancing the role of communities in solving environmental problems.

2. Methods and materials

2.1. Developing indicators, weighting and mapping methods

In our analysis, the eco-environmental vulnerability profile is defined as representation of the current eco-environmental status and considerable natural and manmade disturbances. We first carried out a review of data availability with well-documented global datasets that result in 16 indicators as distributed into five major disturbance determinants including hydrometeorology (B_1); socioeconomics (B_2); land resources (B_3); natural hazards (B_4); and topography (B_5). Table 1 provides a summary of selected indicators, including their sources and roles. All spatial analysis processes were mainly conducted in ArcGIS version 10.3 software in WGS84 projection with pixel size of 0.0833 degree (Fig. 1). The overall framework implemented in this study is shown in Supplementary Fig. S1. As most of the selected indicators have different units and formats, firstly, we converted all of them into the same format with classified range values. In this study, we adopted and modified our previously proposed frameworks Nguyen et al. (2016) and Liou et al. (2017) to become suitable for a global scale study of eco-environmental vulnerability assessment under the condition of global dataset's availability. Note that we enhance our previous frameworks by adding natural hazard disturbance factors associated with climate change. Consequently, the current framework becomes more comprehensive in capturing human-made and natural disturbance footprints on eco-environment. An analytical hierarchy process (AHP) and geographical information system (GIS) were carried to combine multi-indicators in groups and then further aggregated to become one final indicator of GEV by using equations (Adger et al., 2013; Adger, 2006):

$$GEV = \sum_{1}^{5} B_i * W_i \quad (1)$$

$$B_i = \sum_{1}^{nBi} C_j * w_j \quad (2)$$

where GEV denotes the global eco-environmental vulnerability (the higher the GEV value, the greater the vulnerability is likely to be), B_i is the *i*th group determinant factor, W_i is the weight of the *i*th group determinant factor, C_J is the *j*th indicator, w_j is the weight of the *j*th variable, and nB_i is the number of indicators in a group determinant factor B_i introduced in Table 1. Weights of 16 indicators and five groups are presented in the Table 2. To classify vulnerable intensity, the GEV was standardized and compared. In this study, we used histograms to reveal the statistical distribution corresponding to values of grid cells of eco-environmental vulnerability raster to classify GEV assessment into six categories, namely *very low* (<1.66), *low* (1.66–2.13), *medium* (2.13–2.56), *medium high* (2.56–3.06), *high* (3.06–3.59), and *very high* (>3.59) (Fig. 2).

Table 1
Indicators used to evaluate global eco-environmental vulnerability including their sources, data description, preparation, and brief explanation of their roles.

Major disturbance determinants	Indicators	Role in environment profile	Data preparation	Sources
Hydrometeorology (B_1)	Soil moisture (C_1)	Soil moisture is vitally important in controlling the exchange of water and heat energy between land surface and atmosphere through evapotranspiration and as a key variable to define flood control, soil erosion, and slope failure.	Annual average soil moisture was retrieved from L-band microwave missions SMOS (Soil Moisture and Ocean Salinity) as output in NETCDF file, further converted to GEOTIFF format, and then mosaicked and classified into eight classes over a global scale of 0.25-degree grid. Soil moisture is provided by INRA (Institut National de la Recherche Agronomique) and CESBIO (Centre d'Etudes Spatiales de la BIOsphère). We interpolated the areas with insufficient data or data being removed due to unsuitable values of shallow effects.	Moran et al. (2007)
	Precipitation (C_2)	Precipitation is important for soil and plant growth and useful for determination of weather patterns regarding to early warning of drought and flood.	Global annual average precipitation (mm) with a resolution of 0.00833 degree from a GEOTIFF file was classified into eight classes. The precipitation data was interpolated using thin-plate splines with covariates including elevation, distance to the coast. Weather station data were used between 9000 and 60,000 stations with temporal range of 1970–2000 (Ficka and Hijmans, 2017).	Ficka and Hijmans (2017)
	Temperature (C_3)	Average global air temperature is useful to classify weather patterns in combination with precipitation and soil moisture.	Global average temperature with a resolution of 0.00833 degree from a GEOTIFF file was classified into eight classes. The data was processed by using the same weather station and method as precipitation data (Ficka and Hijmans, 2017).	Ficka and Hijmans (2017)
	Distance from hydrological network (C_4)	Availability of surface water is important for environment especially in urban cities for cooling heat island effect.	By using shape file formatted inland water surface data, we calculated the distances of interest by a Euclidean distance tool in ArcGIS, which were then further classified into eight classes.	http://wp.geog.mcgill.ca/hydrolab/
Socioeconomics (B_2)	Population (C_5)	Population plays an important role in eco-environmental vulnerability assessment since it contributes to determine human pressure on eco-environment. In general, more people and higher population density likely cause heavier pressure on environment resulting in higher vulnerability.	Population data are in excel file format, stored into a shape file, and further converted to GEOTIFF format. Finally, population is classified into eight classes.	https://data.worldbank.org
	Income (C_6)	This indicator shows average income of each country from high to low income (highly-developed countries to developing countries). In general, in the developing countries, the eco-environment is likely to be disturbed more than developed countries since they are on the fast growing processes of urbanization and industrialization. Income also reflects the education level as well as public awareness of eco-environmental protection.	Income data are in excel file format, stored into a shape file, and then further converted to GEOTIFF format. Raster of income is classified into five classes.	https://data.worldbank.org
	Distance from urbanized areas (C_7)	This indicator determines the influence from the urban by spatial distance. Exposure from urban affected the eco-environment by the stress from the city like pollution from vehicles and air-condition, and trash from households, and wastewater. It is likely that the farther from the urban the better the eco-environment.	Urban areas are in shape file format. We calculated the distance by a Euclidean distance tool in ArcGIS, which was further classified into eight classes from near to far from the urbanized areas.	http://preview.grid.unep.ch
Land resource (B_3)	Land use/land cover (LULC) (C_8)	LULC is an important determinant of eco-environmental vulnerability due to its contribution to and general influence on environmental quality. The areas without or with less vegetation cover are more vulnerable than the dense vegetation areas. Impervious surface materials conserve more heat during the day and release it more slowly at night than natural materials like soil or vegetation[52].	The MODIS Land Cover Type product in 2017 (Short Name: MCD12Q1) of 500 m SIN Grid in HDF format were download and further processed such as mosaicked and converted into GEOTIFF format and then classified into 16 classes following the legend and instruction of providers. References include USGS's website and following papers (Friedl et al., 2002; Latham et al., 2014).	https://reverb.echo.nasa.gov https://landcover.usgs.gov/glc/
	Normalized Difference Vegetation Index (NDVI) (C_9)	NDVI is a crucial indicator to measure the greenness of vegetation and vegetation plays an important role in maintaining good eco-environment[53]. Regions that are less or without vegetation may cope with higher vulnerability.	Global MODIS vegetation indices, NDVI product Vegetation Indices 16-Day L3 Global 500 m were downloaded, mosaicked, and used to compute mean value NDVI of the year 2017, and then further classified into five classes.	https://reverb.echo.nasa.gov
Natural hazards (B_4)	Drought (C_{10}) Tropical cyclones (C_{11}) Landslides (C_{12}) Flood (C_{13})	These indicators determine the areas constantly affected by natural hazards resulting in environmental decline.	These indicators are downloaded either in excel file, shape file or raster GEOTIFF format, then further processed into same format of GEOTIFF at resolution of 0.0833 degree over a global scale, and aggregated by weighted sum function in ArcGIS (each type of natural hazard has the same weight). Final raster of natural hazards is classified into five classes.	Global Risk Data Platform http://preview.grid.unep.ch
Topography ($B5$)	DEM (C_{14})	DEM plays an important role in defining topographic condition, determining the features of land surface such as incoming solar radiation, tree types, and potential exposure to hazards like landslide, and drought.	SRTM DEM in GEOTIFF file was downloaded with a resolution of 0.00833 degree and further processing was conducted such as filling holes, and classification into eight classes.	http://glcf.umd.edu/data/srtm/
	Slope constraint	Slope constraint is a factor influencing land-use decision and the item "Land utilization possibilities". The influence	Slope constraint with 0.0833 degree resolution was downloaded and converted into GEOTIFF file and	http://www.fao.org/geonetwork

(continued on next page)

Table 1 (*continued*)

Major disturbance determinants	Indicators	Role in environment profile	Data preparation	Sources
	(C_{15})	of terrain on erosion is great important[56]. Steeper slopes are also associated with shallower soils in general and with a higher risk for soil degradation and landslides (Aksoy and Kavvas, 2005; Eliasson et al., 2010; Böttcher et al., 2009).	classified into 8 classes from low constraint to very frequent constraint.	
	Slope aspect (C_{16})	Slope aspect and topographic position contribute to define annual mean temperature, potential energy incoming and evapotranspiration. Resulting in vegetation structure, ground moisture, snow retention, plant communities and surface temperature are all characteristics influenced by aspect (Viles and Cutler, 2012).	Slope aspects were computed by using ArcGIS function with input SRTM DEM and then further classified into 10 classes.	SRTM DEM http://glcf.umd.edu/data/srtm/

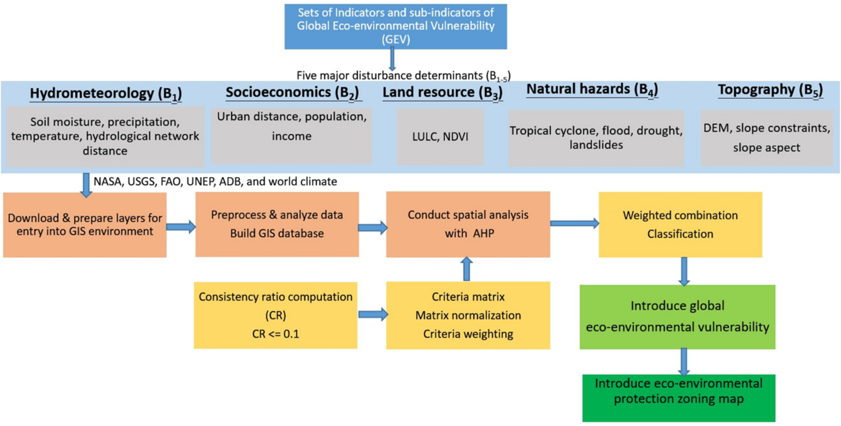

Fig. 1. Framework for the global eco-environmental vulnerability assessment. LULC is land use/land cover; NDVI is normalized difference vegetation index; and AHP is analytical hierarchy process.

3. Results and discussion

3.1. Global and continental vulnerability distribution

A global eco-environmental vulnerability (GEV) map is presented in Fig. 2. GEV hotspots for various continents are highlighted in A, B, C, D, E, and F, primarily locating in Asia, east Africa, and east North America. Fig. 3 presents a histogram of accumulated disturbance scores depicting the fraction of global areas that falls within each vulnerability level.

Table 2
Weightings of group indicators and indicators used for the calculation of global eco-environmental vulnerability (modified and adapted from Böttcher et al., 2009; Drought Monitoring Centre (DMC), 2000). Consistency ratio of assessment is 0.007. Class weights and consistency ration of each indicator are provided in Table S4.

Group variables/factors (B_i)	Global weight (W_i)	Variables/factors (C_j)	Local weight (w_j)
B_1. Hydrometeorology	0.169	C_1 Soil moisture	0.384
		C_2 Precipitation	0.300
		C_3 Temperate	0.191
		C_4 Distances from hydrological network	0.125
B_2. Society-economics	0.242	C_5 Population	0.557
		C_6 Income	0.320
		C_7 Distances from urbanized areas	0.123
B_3. Land resources	0.070	C_8 LULC	0.667
		C_9 NDVI	0.333
B_4. Natural hazards	0.395	C_{10} Drought	0.250
		C_{11} Tropical cyclone	0.250
		C_{12} Landslide	0.250
		C_{13} Flood	0.250
B_5. Topography	0.123	C_{14} DEM	0.557
		C_{15} Slope constraint	0.320
		C_{16} Slope aspect	0.123

Distribution represents the percentage of each vulnerability category based on number of pixels (1 pixel = 0.083 degree). Fig. 4 and Table 3 give estimated distribution of vulnerability in various continents.

It can be obviously seen from Figs. 2 and 3 that about half of global land exhibits in *very low* and *low* vulnerability levels (around 49.2% of the globe). They mainly appear in the high-latitude (59.8% of *very low* vulnerable level attributed to North America, and 11.7% and 38.5% of *very low* and *low* vulnerability levels attributed to Europe as listed in Table 3) where are seasonally covered by ice or land types of grassland or shrub land and forest (Supplementary Fig. S1). Many parts of these areas are natural conservation areas with less manmade disturbances. However, under the circumstance of increased global temperature and ice smelting, eco-environment of these regions will be significantly influenced in the future. The *medium* and *medium high* vulnerability levels occupy approximately 40.7% of the globe, while they are intensively distributed in Asia, Europe, Africa, and a tiny part in North America (Figs. 2, 4 and Table 3). These regions are seasonally barren land or grassland with less vegetation cover and under drastic weather so that they are likely drought vulnerable sectors (Barlow et al., 2002; Brooks, 2004; DMC, 2000; Calliham et al., 1994; UNEP, 2002). In addition, these regions might experience consequences of economic activities with greenhouse gases and human access as significant disturbance factors. Impressively, South Asia, East Africa, and a tiny part of North America are of *high* and *very high* vulnerability levels, approximately 10.1% of the globe. *High* vulnerability level is significantly attributed to Asia by about 55.3%, followed by Africa 19.0%, and North America 10.9%; *very high* vulnerability is most distributed in Asia by 74.6% followed up by Africa's 19.6% and North America's 3.2% (Table 3).

Our analysis shows that there are various factors causing seriously environmental vulnerability in Asia, Africa, and a tiny part of North America (Fig. 4 and Table 3 with hotspots highlighted in A, B, C, D, E, and F in red squares). Why does North America still exist in *high* and *very high* vulnerabilities? The answer might have been given in the

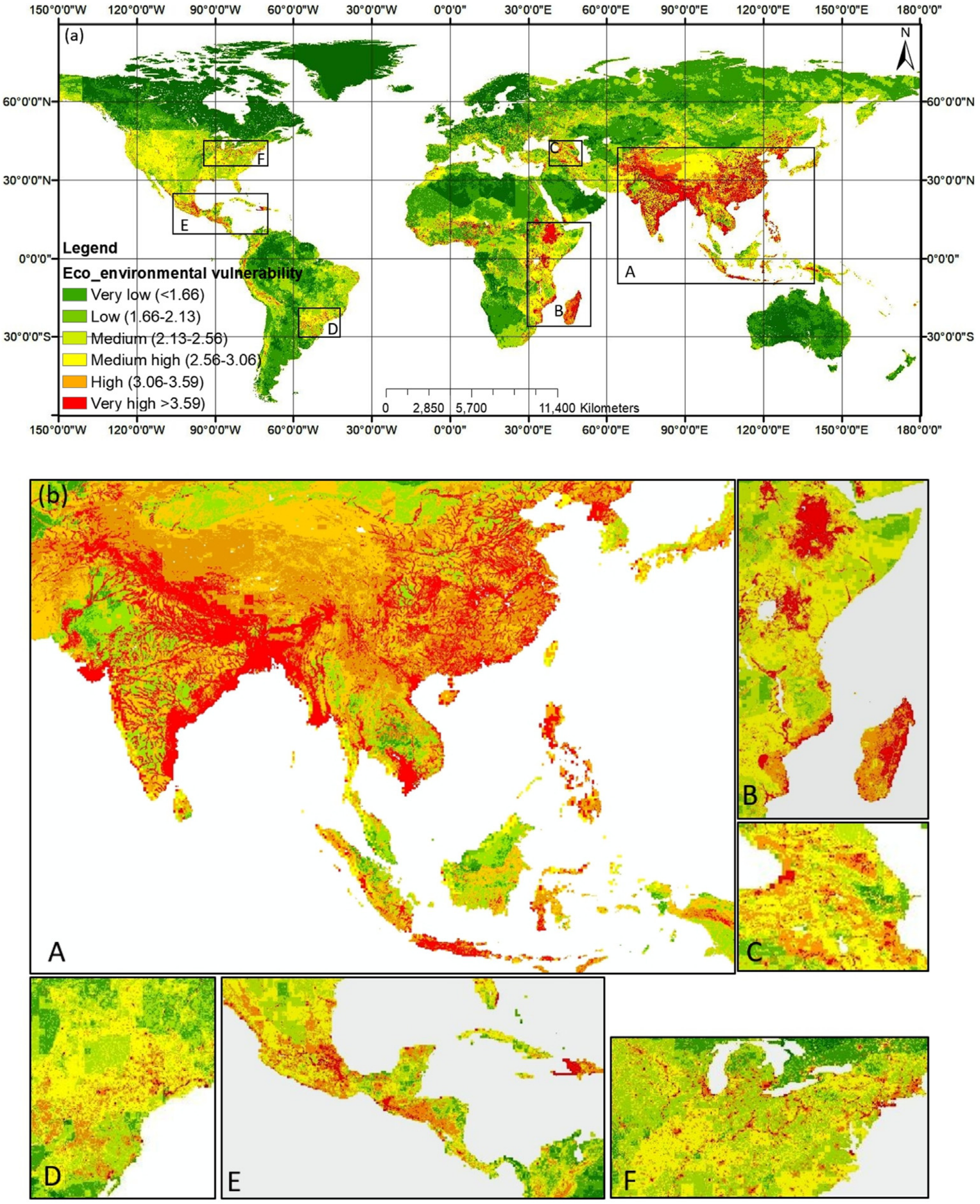

Fig. 2. (a) A global eco-environmental vulnerability map and (b) its hotspots highlighted in A, B, C, D, E, and F for various continents.

literature in that the disturbances were caused by on-going climate warming in arctic, subarctic, and boreal environments (Ford et al., 2015; Hinzman et al., 2005). In addition, previous studies indicated that northern high latitudes would be affected by global climate change earlier and stronger than the other regions of the earth (IPCC, 2007). Consequently, strong dynamic feedbacks and interactions between northern high-latitude biosphere and atmosphere are an important driving force of climate chance with teleconnections to lower latitudes (Serreze and Francis, 2006; McGuire et al., 2006). In addition, North America, followed by Asia, exposed the highest rate of forest cover loss caused by Agro-industrial clearing, selective logging, fire, and diseases (Hassan et al., 2010). Firstly, the fact cannot be declined that many countries in *high* and *very high* vulnerable levels are under developing process (Fig. 5). As a result, many processes of socioeconomic and anthropogenic disturbances cause burden for environment.

Secondly, with huge population and its continuous growth at a high rate resulted in an increased demand of replacing natural land by intensive agricultural (Jeong et al., 2014; Valipour, 2017) land and urbanized land, inducing a heavier pressure on environment. Thirdly, it cannot be declined that these regions are most suffered from natural hazards, such as flood, drought, landslides, sea level rise, heatwave, and especially tropical cyclones (Woods, 1989; Dao and Liou, 2015) (Supplementary Fig. S6 (d)). However, in this study, we did not take into account all kinds of natural hazards that may happen in these regions such as earthquake, forest fires, and volcanic eruption before we are able to find robust datasets even though they may be of high impacts on eco-environment. Finally, the situations of low income in general, fast economic development, and correspondingly lack of access to good education result in low awareness of households in these regions in terms of environmental protection (Yohe and Tol, 2002).

3.2. National-scale distribution of vulnerability

Top ten countries in Asia that have either *high* or *very high* vulnerability level exceeding 1000 pixels include China, India, Pakistan, Indonesia, Myanmar, Nepal, Bangladesh, Afghanistan, Vietnam, and Thailand (Fig. 6). Note that it would be much easier for bigger countries to hit the threshold of the 1000 pixels in the higher classes, while, for

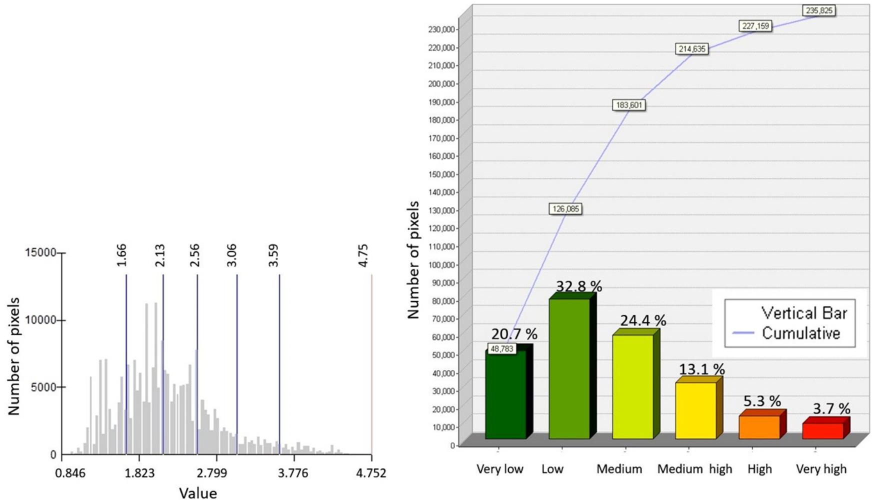

Fig. 3. Histogram of accumulated disturbance scores depicting the fraction of global areas that falls within each vulnerability level. Distribution represents the percentage of each vulnerability category based on the number of pixels.

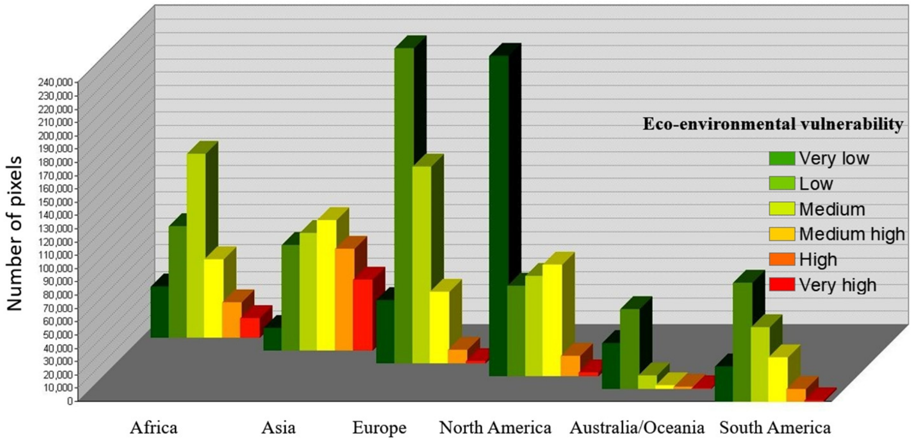

Fig. 4. Estimated distribution of GEV by continents. (For interpretation of the references to color in this figure, the reader is referred to the web version of this article.)

obvious statistical reasons, it would be more difficult for smaller countries, even if the relative distribution of the vulnerability classes are the same. Our analysis shows that China and India are the most vulnerable in Asia and in the world with 20,912 pixels in *very high* level and 41,250 pixels in *high* level in China followed by India with 16,943 pixels in *very high* level and 5507 pixels in *high* level. Following Asia, Africa is the continent that has the second most *very high* and *high* vulnerability levels with other top vulnerable countries including Ethiopia, Madagascar, Kenya, Nigeria, Mozambique, Tanzania, and Congo. Ethiopia and Madagascar are the most vulnerable countries in Africa with 3898 pixels and 2316 pixels in *very high* vulnerability level, respectively. In America, USA, Mexico, Brazil, Peru, and Colombia are the leading countries suffering the most vulnerability. Perhaps not surprisingly, anthropogenic disturbances linked to physical size of population, and fast economic development incorporated with high intensity devastating impacts from natural hazards (Supplementary Fig. S4 and Fig. S6) lead South Asia and East Africa countries to be the most vulnerable regions in the world (Kan et al., 2012). It is vital that eco-environment of these regions will need more special care in particular under the situation of global climate change with more frequent and

Table 3
Percentage of vulnerabilities at continental scale.

GEV	Africa	Asia	Europe	North America	Oceania	South America
**Very low**	9.4	4.0	**11.7**	**59.8**	8.4	6.5
**Low**	13.6	12.8	38.5	11.0	9.7	14.5
**Medium**	**26.9**	17.1	**28.7**	14.5	1.9	10.8
**Medium high**	17.7	**29.8**	16.3	25.4	0.7	10.1
**High**	**19.0**	**55.3**	**7.1**	10.9	0.9	6.8
**Very high**	**19.6**	**74.6**	**1.8**	3.2	0.1	0.8

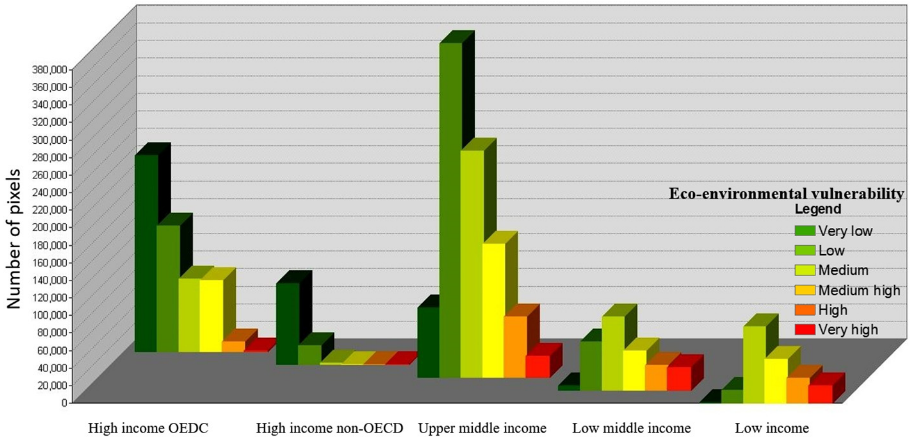

Fig. 5. Estimated distribution of GEV by countries' income category.

unpredictable natural hazards. Our findings show a good link and are in line with outcomes from the other researches. Halpern et al. (2008) and Fargione et al. (2008) found the areas where are *high* and *very high* vulnerable of human impacts are commonly seen among the contributing factors causing pressure on environment of inland and marine. IPCC (2001) and Ericson et al. (2006) indicated that intensive disturbances from anthropogenic stress enhanced by natural hazards bring more stress on environment.

3.3. Uncertainty estimates

To estimate the confidence level of global eco-environmental vulnerability map, we based on the estimation of the uncertainty level of the input datasets and mapping procedure (Tables S2 and S3). The confidence level was divided into five categories, namely highest, high, moderate, low, and unknown as listed in Table S6. The confidence is rated according to the level of confidence about the generated mapping procedure and spatial datasets used (Table S7). This approach provides reliable outputs to validate our eco-environmental vulnerability assessment, justifying the confidence for data and mapping procedures. That is, the eco-environmental vulnerability assessment was rated as high level of confidence since the dataset used were derived from good quality inputs with peer reviewed methods. For example, the soil moisture data and natural hazard events were rated as the highest confidence. Soil moisture data generated from SMOS satellite mission has been validated by field survey. In contrast, social economics data and hydro meteorological data were rated as high confidence level for their clearly published sources and being generated from in situ instruments, respectively, while topological data and land use/land cover indicators were rated as moderate confidence level since they were derived from SRTM and MODIS data, respectively. To recap, all of the datasets used for global eco-environmental vulnerability mapping were rated as high confidence because they were derived from high quality data with peer reviewed methods except NDVI rated as moderate confidence due to the fact that they are MODIS products. As for the mapping procedures, they were well accepted because they were done by a common practice by using ArcGIS software package.

3.4. Validation

Validation of the global EVA map results is necessary to promote the value of the proposed framework in this paper, while tremendous

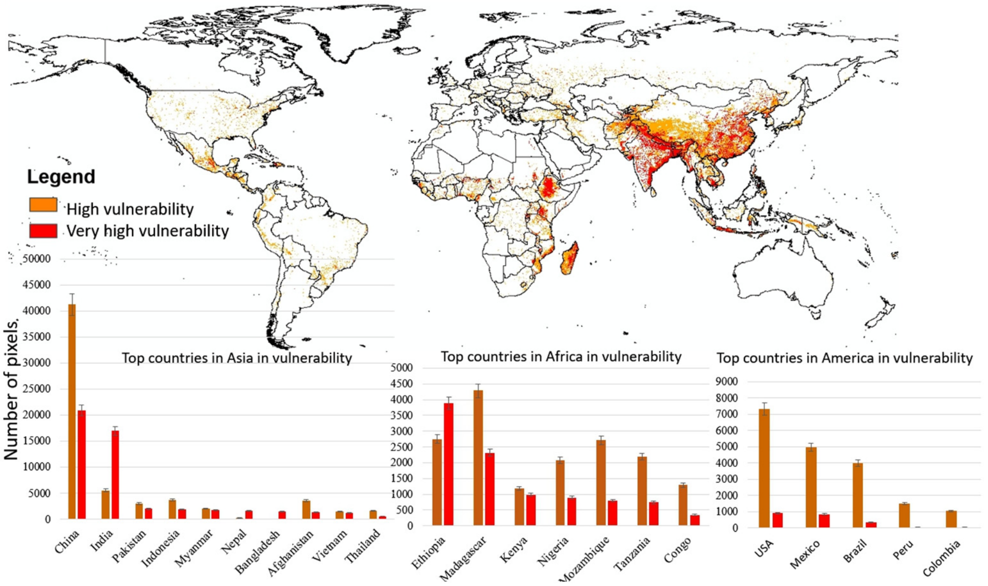

Fig. 6. Estimated *high* and *very high* vulnerability levels in continents with top suffering countries (error bars represent 95% confidence intervals for the areas of *high* and *very high* vulnerability).

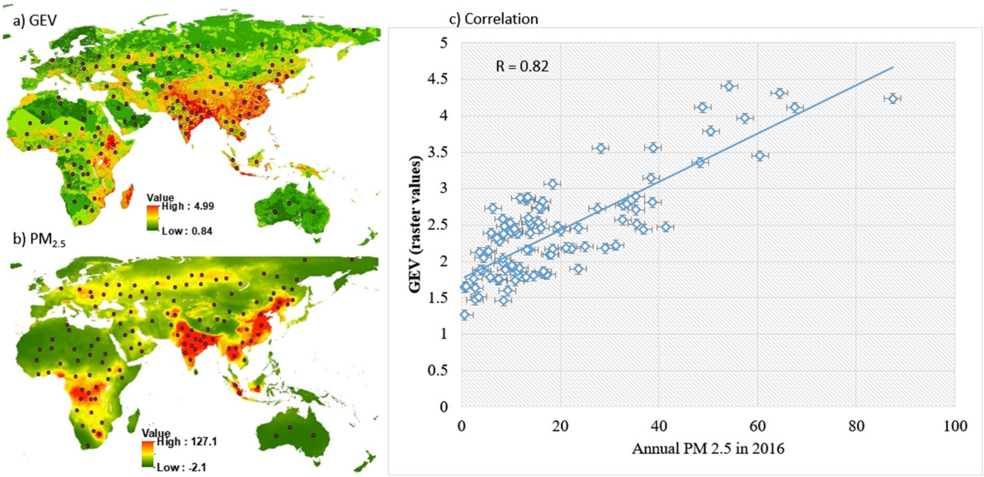

Fig. 7. Comparison of (a) the global eco-environmental vulnerability map with (b) annual $PM_{2.5}$ distribution in 2016. (c) Correlation coefficient is 0.82 for 100 randomly chosen checking points over the globe.

efforts beyond the scope of this paper will be required to implement a detailed validation about the results. Alternatively, we attempted an initial validation about the results, and leave the detailed validation for further studies. Thus, an independent variable $PM_{2.5}$ that can be considered as an anthropogenic disturbance associated with nature and human-made influence was chosen to validate the GEV map. A global map of $PM_{2.5}$ was derived from MODIS data (van Donkelaar et al., 2016, 2018). One hundreds of pixels were then randomly chosen from the global map for analysis of correlations between EVA map results and $PM_{2.5}$ pollution distribution. It is found that the correlation coefficient reaches 0.82 as shown in Fig. 7. Such a reasonably high correlation supports the reliability of the EVA map even though further validation is still required.

3.5. Advised eco-zones for eco-environmental protection

FAO (2001) provided a map of ecological zones by taking into account the influence of agriculture, forestry, fishery and food security for decision making. Here we introduced three eco-environmental zones subject to global environmental policy and management as shown in Fig. 8. In addition, the eco-environmental baselines, which determine the importance of ecological features, present characteristics of specific regions for design options to avoid significant impacts on important ecological features/resources at an early stage. Note that most, if not all, land planning will require an eco-environmental impact assessment. Thus, the advised eco-environmental baselines would serve as basis of the first step to inform a developer of the key eco-environmental status, design options and requirements for further investigation and mitigation measures. How detail further eco-environmental surveys will depend on the features of specific regions, and purposes of developments and regulators. Three eco-environmental zones are described in the following: *(i)* Eco-zone I (EZ-I) (combined areas of *very low* and *low* vulnerability levels) is for *focal protection,* which is mainly characterized by identification land, natural park and conservation areas. It is suggested not to encourage any further development in these areas. *(ii)* Eco-zone II (EZ-II) (combined areas of *medium* and *medium high* vulnerability levels) is for *composite development* that is mixed with living environment and natural space. Thus, it is more complicated to maintain and enhance the functional ecosystem. To retain and enhance the visual scenic qualities of the landscape and high ecological values, environment protection is required. Additionally, intensive agriculture and heavy industry are not

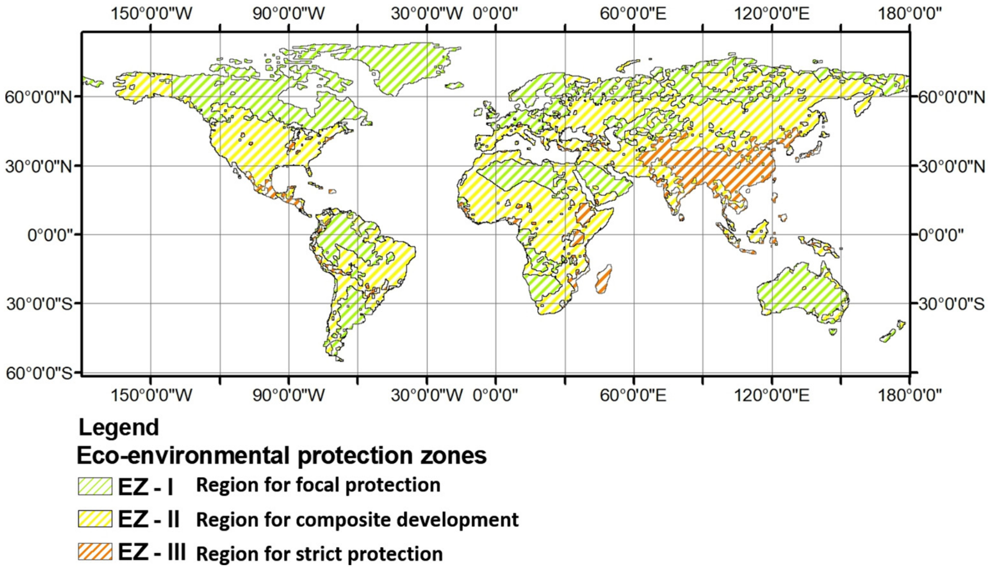

Fig. 8. The map of proposed eco-environmental protection zones.

advised to develop in these regions. *(iii)* Eco-zone III (EZ-III) (combined areas of *high* and *very high* vulnerability levels) is for *strict protection*. It is needed for a limited range of development in urbanization, industrialization, and intensive agricultural practice to reduce the impacts of residential development in the areas and ensure residential development not to have a diversified effect on eco-environment. Environmental protection works are crucial here to control access to forestry, limit appropriate agriculture activities, and enhance green industry to improve environmental facilities and restore the ecological values. All of these aim to improve the natural eco-environment and protect it from being threatened or affected by natural or manmade disturbances, such as aggrieve or unsustainable farming and logging, pollution on the soil in the cultivated areas, urban expansion or sprawl due to increased population. However, implementation of the said activities will face various challenges and debates during the transition phase among beneficial groups, such as local communities with access rights, businessmen with development interests, government officials, and people of environmentalism.

4. Conclusion

We proposed an approach to estimate GEV based on main disturbance sources, either natural or manmade. A global map of eco-environmental vulnerability profile was generated by synthesizing 16 influential indicators and classified into six levels. Our assessment framework can be used to identify manmade and natural impacts on eco-environment. It opens an opportunity to look at a wide range of scales, from local, regional to global scales where higher and better quality of satellite images or data products are available with good weather condition such as Copernicus Sentinel-2 imagery in order to construct different environmental variables. It would be even better to further develop validation sites of this approach over regional or local scale. Overall speaking, the assessment framework and selected indicators can be proposed in many approaches. Our study introduced a clear formulation and description of data used. However, the outcomes are still subject to certain uncertainties linking to diversified global datasets of different sources and analysis concepts. This is because each dataset has its own limitation and uncertainty, leading to accumulative uncertainty. Furthermore, a global view with top-down overview may lead to over simplified conclusions. It would be helpful to combine with local information to utilize the outcomes of this study for decision making.

Despite the abovementioned limitations, our results bring benefits to environmental management and conservation on land from a global view that has not been found in the literature as far as we know. To highlight the accumulative impacts of human and nature on eco-environment and provide the best evaluation of the current global homogeneous map in terms of eco-environment vulnerability, three points are stressed. *(i)* Human and nature bring more pressure on eco-environment in Asia and Africa because in these regions human beings significantly depend on goods and services from ecosystems and natural resources. The largest fraction of *very high vulnerability* level is attributed to Asia (74.6%) followed by Africa (19.6%). The pressure will continue to increase with a high rate of growing population and intensive infrastructure development. In addition, with a long history of overgrazing and cultivating, and intensive agriculture practice in less developed regions like Asia and Africa lead soil degradation and erosion "The least developed economies have been found to experience the highest estimates of soil erosion rates. The greatest increases are predicted to occur in Sub-Saharan Africa, South America and Southeast Asia" (Borrelli et al., 2013). Moreover, due to topographic features incorporated with climate change effects, Asia and Africa are significantly influenced by frequent natural hazards, leading to enhanced disturbances. *(ii)* Advised eco-environmental zones are introduced to assist the decision makers with specific strategies to lessen the negative impacts of manmade and natural disturbances, which can in turn benefit both human and nature. Frankly speaking, natural disturbances may be resulted from human activities so that effective measures to mitigate natural hazards should start from regulating human activities. For instance, enhancing urban greenspaces infrastructure is needed to maximize their eco-system services and, subsequently, to reduce air pollution and summer heatwaves (Nguyen et al., 2018). Therefore, it is of highest priority environmental police to encourage the people to adopt eco-environmental friendly materials and green transportation to reduce greenhouse gases emission. In the meantime, there is a need to enhance the sustainable environment in the industrialization zone and improve natural resource awareness by training the people especially in the tropical rainforest region like Indonesia, Malaysia, Congo where always face with forest logging and forest fire, industrial agricultural practices, etc. *(iii)* It would be important to use higher resolution datasets for the identification of hotspot areas like Asia and Africa to find more details of causes with time series evaluation to reveal the changes and characteristics of their driving forces.

Author contributions statement

A.K.N and Y.A.L came up with research idea through discussion. A.K.N conducted data collection, data processing, and data analysis with assistance from Y.A.L. A.K.N prepared a draft manuscript and Y.A.L enriched and finalized the manuscript.

Competing interests

The authors declare no conflict of interest.

Acknowledgments

This research was financially supported by Ministry of Science and Technology (MOST) of Taiwan under the codes 105-2221-E-008-056-MY3, 107-2111-M-008-036, and 107-2622-E-008-006-CC3, United States Geological Survey (USGS), World Bank, and FAO support global datasets. The authors express sincere thanks to Prof. Jean-Pierre Wigneron and his team (Dr. Amen Al-Yaari) for providing Global Soil Moisture Products, and the three anonymous reviewers for providing constructive comments to improve the paper.

Appendix A. Supplementary data

Supplementary data to this article can be found online at https://doi.org/10.1016/j.scitotenv.2019.01.407.

References

Adger, W.N., 2006. Vulnerability. Glob. Environ. Chang. 16 (3), 268–281.
Adger, W.N., Barnett, J., Brown, K., Marshall, N., O'Brien, K., 2013. Cultural dimensions of climate change impacts and adaptation. Nat. Clim. Chang. 3, 112–117.
Aksoy, H., Kavvas, M.L., 2005. A review of hillslope and watershed scale erosion and sediment transport models. Catena 64, 247–271. https://doi.org/10.1016/j.catena.2005.08.008.
Aretano, R., et al., 2017. Mapping ecological vulnerability to fire for effective conservation management of natural protected areas. Ecol. Model. 295, 163–175.
Barlow, M., Cullen, H., Lyon, B., 2002. Drought in central and southwest Asia: La Niña, the warm pool, and Indian Ocean precipitation. J. Clim. 15 (7), 697–700.
Berrouet, L.M., Machado, J., Palacio, C.V., 2018. Vulnerability of socio—ecological systems: a conceptual framework. Ecol. Indic. 84, 632–647.
Borrelli, B., et al., 2013. An assessment of the global impact of 21st century land use change on soil erosion. Nat. Commun. 8. https://doi.org/10.1038/s41467-017-02142-7.
Böttcher, K., et al., 2009. Guidelines for application of common criteria to identify agricultural areas with natural handicaps. European Communities. EUR 23795 EN.
Brooks, N., 2003. Vulnerability: risk and adaptation: a conceptual framework. Tyndall Centre Clim. Change Res. Working Pap. 38, 1–16.
Brooks, N., 2004. Drought in the African Sahel: long-term perspectives and future prospects. Tyndall Centre for Climate Change Research. University of East Anglia (Working paper).
Calliham, D.M., Eriksen, J.H., Herrick, A., Famine, B., 1994. The United States government response to the 1991/92 Southern Africa drought. Evaluation Synthesis Report. Management Systems International, Washington.

Dao, P.D., Liou, Y.A., 2015. Object-based flood mapping and affected rice field estimation with Landsat 8 OLI and MODIS data. Remote Sens. 7 (5), 5077–5097. https://doi.org/10.3390/rs70505077.

De Lange, Sala, H.J., Vighi, S.M., Faber, J.H., 2010. Ecological vulnerability in risk assessment - a review and perspectives. Sci. Total Environ. 408, 3871–3879. https://doi.org/10.1016/j.scitotenv.2009.11.009.

Drought Monitoring Centre (DMC), 2000. Drought Monitoring Centre Dekad 19 report. *Ten day bulletin* No. DMCN/01/337/19/07.

Eakin, H., Luers, A.L., 2006. Assessing the vulnerability of social-environmental systems. Annu. Rev. Environ. Resour. 31, 365–394. https://doi.org/10.1146/annurev.energy.30.050504.144352.

Eitner, B.M.A., 2016. Ecological vulnerability indicators. Ecol. Indic. 60, 329–334. https://doi.org/10.1016/j.ecolind.2015.07.001.

Eliasson, A., et al., 2010. Common criteria for the redefinition of intermediate less favoured areas in the European Union. Environ. Sci. Pol. 13, 766–777. https://doi.org/10.1016/j.envsci.2010.08.003.

Ericson, J.P., Vorosmarty, C.J., Dingman, S.L., Ward, L.G., Meybeck, M., 2006. Effective sea-level rise and deltas: causes of change and human dimension implications. Glob. Planet. Chang. 50, 63–82.

FAO, 2001. Global Ecological Zones, spatial database of global ecological zones Food and Agriculture Organization of the United Nations (FAO). http://www.fao.org/geonetwork Accessed 9 Apr 2009. (2001).

Fargione, J., Hill, J., Tilman, D., Polasky, S., Hawthorne, P., 2008. Land clearing and the biofuel carbon debt. Science 319 (5867), 1235–1238. https://doi.org/10.1126/science.1152747.

Fawcett, D., Pearce, T., Ford, J.D., Archer, L., 2017. Operationalizing longitudinal approaches to climate change vulnerability assessment. Glob. Environ. Chang. 45 (Suppl. C), 79–88.

Ficka, S.E., Hijmans, A.J., 2017. WorldClim 2: new 1-km spatial resolution climate surfaces for global land areas. Int. J. Climatol. https://doi.org/10.1002/joc.5086 (2017 http://worldclim.org/version2.

Ford, J.D., McDowell, G., Pearce, T., 2015. The adaptation challenge in the Arctic. Nat. Clim. Chang. 5, 1046–1053.

Friedl, M.A., et al., 2002. Global land cover mapping from MODIS: algorithms and early results. Remote Sens. Environ. 83 (1–2), 287–302.

Garnier, A., Pennekamp, F., Lemoine, M., Petchey, O.L., 2017. Temporal scale dependent interactions between multiple environmental disturbances in microcosm ecosystems. Glob. Chang. Biol. 23, 5237–5248.

Halpern, B.S., et al., 2008. A global map of human impact on marine ecosystems. Science 319 (5865), 948–952. https://doi.org/10.1126/science.1149345.

Hassan, M.C., Stehman, S.V., Potapov, P.V., 2010. Quantification of global gross forest cover loss. PNAS 107 (19), 8650–8655.

Hinkel, J., 2011. Indicators of vulnerability and adaptive capacity: towards a clarification of the science-policy interface. Glob. Environ. Chang. 21, 198–208. https://doi.org/10.1016/j.gloenvcha.2010.08.002.

Hinzman, L.D., et al., 2005. Evidence and implications of recent climate change in northern Alaska and other arctic regions. Clim. Chang. 72 (3), 251–298. https://doi.org/10.1007/s10584-005-5352-2.

http://glcf.umd.edu/data/srtm/

http://preview.grid.unep.ch

http://wp.geog.mcgill.ca/hydrolab/

http://www.fao.org/geonetwork

https://data.worldbank.org

https://landcover.usgs.gov/glc/

https://reverb.echo.nasa.gov

IPCC, 2001. Intergovernmental Panel on Climate Change: Climate Change: Impacts, Adaptation and Vulnerability. Cambridge University Press, Cambridge.

IPCC, 2007. The physical science basis. In: Solomon, S., et al. (Eds.), Contribution of Working Group I to the Fourth Assessment Report of the *Intergovernmental Panel on Climate Change*. Cambridge Univ. Press, New York.

Ippolito, A., Sala, S., Vighi, M., 2010. Ecological vulnerability analysis: a river basin case study. Sci. Total Environ. 408 (18), 3880–3890.

Janssen, M., Ostrom, E., 2006. Special issue on resilience, vulnerability, and adaptation: a crosscutting theme of the human dimensions of global environmental change program. Glob. Environ. Chang. 16, 237–239.

Jeong, S.J., et al., 2014. Effects of double cropping on summer climate of the North China plain and neighboring regions. Nat. Clim. Chang. 4, 615–619.

Johnson, J.E., et al., 2016. Assessing and reducing vulnerability to climate change: moving from theory to practical decision-support. Mar. Policy 74, 220–229. https://doi.org/10.1016/j.marpol.2016.09.024.

Jongman, et al., 2015. Declining vulnerability to river floods and the global benefits of adaptation. PNAS 112 (18), E2271–E2280.

Kan, H., Chen, R., Tong, S., 2012. Ambient air pollution, climate change, and population health in China. Environ. Int. 42, 10–19.

Kumar, P., Geneletti, D., Nagendra, H., 2016. Spatial assessment of climate change vulnerability at city scale: a study in Bangalore, *India*. Land Use Policy 58, 514–532. https://doi.org/10.1016/j.landusepol.2016.08.018.

Lambin, E.F., et al., 2001. The causes of land-use and land-cover change: moving beyond the myths. Glob. Environ. Chang. 11, 261–269.

Latham, J., Cumani, R., Rosati, I., Bloise, M., 2014. *Global Land Cover* SHARE (GLC-SHARE) database Beta-Release Version 1.0. FAO, Rome.

Liou, Y.A., Nguyen, A.K., Li, M.H., 2017. Assessing spatiotemporal eco-environmental vulnerability by Landsat data. Ecol. Indic. 80, 52–65. https://doi.org/10.1016/j.ecolind.2017.04.055.

McGuire, A.D., Chapin, F.S., Walsh, J.E., Wirth, C., 2006. Integrated regional changes in arctic climate feedbacks: implications for the global climate system. Annu. Rev. Environ. Resour. 6 (31), 61–91. https://doi.org/10.1146/annurev.energy.31.020105.100253.

Metzger, M.J., Leemans, R., Schröter, D., 2005. A multidisciplinary multi-scale framework for assessing vulnerabilities to global change. Int. J. Appl. Earth Obs. Geoinf. 7, 253–267. https://doi.org/10.1016/j.jag.2005.06.011.

Moran, R.F., et al., 2007. SMOS-IC: an alternative SMOS soil moisture and vegetation optical depth product. Remote Sens. 9, 457. https://doi.org/10.3390/rs9050457.

Nguyen, A.K., Liou, Y.A., Li, M.H., Tran, T.A., 2016. Zoning eco-environmental vulnerability for environmental management and protection. Ecol. Indic. 69, 100–117. https://doi.org/10.1016/j.ecolind.2016.03.026.

Nguyen, T.H., Liou, Y.A., Nguyen, A.K., Sharma, R.C., Phien, T.D., Liou, C.L., Cham, D.D., 2018. Assessing the effects of land-use types in surface urban heat islands for developing comfortable living in Hanoi City. Remote Sens. 2018 (10), 1965.

Rajesh, S., Jain, S., Sharma, P., 2018. Inherent vulnerability assessment of rural households based on socio-economic indicators using categorical principal component analysis: a case study of Kimsar region, Uttarakhand. Ecol. Indic. 85, 93–104.

Raufirad, V., Heidari, Q., Hunter, R., Ghorbani, J., 2018. Relationship between socioeconomic vulnerability and ecological sustainability: the case of Aran-V-Bidgol's rangelands, Iran. *Ecol. Indic*. 85, 613–623.

Raúl, et al., 2017. Assessing forest vulnerability to climate warming using a process-based model of tree growth: bad prospects for rear-edges. Glob. Chang. Biol. 23, 2705–2719. https://doi.org/10.1111/gcb.13541.

Rosa, E.A., Dietz, T., 1998. Climate change and society: speculation, construction and scientific investigation. Int. Sociol. 13, 421–455.

Savo, V., et al., 2016. Observations of climate change among subsistence-oriented communities around the world. Nat. Clim. Chang. 6, 462–473.

Serreze, M.C., Francis, J.A., 2006. The Arctic amplification debate. Clim. Chang. 76, 241–264. https://doi.org/10.1007/s10584-005-9017-y.

Stern, P.C., 1993. A second environmental science: human-environment interactions. Science 260, 1897–1899.

Turner et al., 2003. A framework for vulnerability analysis in sustainability science. PNAS 100 (14), 8074–8807.

United Nations Environmental Programme (UNEP), 2002. United Nations Environmental Programme Africa environment outlook Past, present and future perspectives, *Published to web by GRID-Arendal in Nairobi, Kenya*. (ISBN 92-807-2101-1) 422 pp: UNEP.

Valipour, M., 2017. Global experience on irrigation management under different scenarios. J. Water Land Dev. 32, 95–102. https://doi.org/10.1515/jwld-2017-0011.

van Donkelaar, A., Martin, R.V., Brauer, M., Hsu, N.C., Kahn, R.A., Levy, R.C., Lyapustin, A., Sayer, A.M., Winker, D.M., 2016. Global estimates of fine particulate matter using a combined geophysical-statistical method with information from satellites, models, and monitors. Environ. Sci. Technol. 50 (7), 3762. https://doi.org/10.1021/acs.est.5b05833.

van Donkelaar, A., Martin, R.V., Brauer, M., Hsu, N.C., Kahn, R.A., Levy, R.C., Lyapustin, A., Sayer, A.M., Winker, D.M., 2018. Global Annual PM2.5 Grids from MODIS, MISR and SeaWiFS Aerosol Optical Depth (AOD) with GWR, 1998–2016. NASA Socioeconomic Data and Applications Center (SEDAC), Palisades NY https://doi.org/10.7927/H4ZK5DQS. Accessed DAY MONTH YEAR.

Viles, H.A., Cutler, A.N., 2012. Global environmental change and the biology of heritage structures. Glob. Chang. Biol. 18, 2406–2418. https://doi.org/10.1111/j.1365-2486.2012.02713.x.

Webster, P.J., Holland, G.J., Curry, J.A., Chang, H.R., 2005. Changes in tropical cyclone number, duration, and intensity in a warming environment. Science 309, 1844–1846.

Woods, P., 1989. Effects of logging, drought, and fire on structure and composition of tropical forests in Sabah, Malaysia. Biotropica 21 (4), 290–298.

Yohe, G., Tol, R.S.J., 2002. Indicators for social and economic coping capacity: moving toward a working definition of adaptive capacity. Glob. Environ. Chang. 12, 25–40.
